# Evaluation of vision transformers and vision foundation models for sperm morphology analysis

**DOI:** 10.3389/frph.2026.1883326

**Published:** 2026-07-23

**Authors:** Rawan AlSaad, Shima Albasha, Hasan Burjaq, Moza AlBader, Rajat Thomas

**Affiliations:** 1 AI Center for Precision Health, Weill Cornell Medicine-Qatar, Doha, Qatar; 2 Division of Reproductive Medicine, Hamad Medical Corporation, Doha, Qatar

**Keywords:** artificial intelligence, fertility, foundation model, semen analysis, sperm morphology, vision transformer, medical imaging

## Abstract

**Background:**

Sperm morphology assessment is an essential but highly variable component of semen analysis, influenced by staining, image quality, acquisition conditions, and observer interpretation. Modern pretrained visual encoders may improve the objectivity and reproducibility of this assessment, but their performance and robustness in sperm morphology analysis remain insufficiently characterized.

**Objectives:**

To compare vision transformers and vision foundation models with established pretrained CNNs for sperm morphology classification; assess the effect of adaptation strategy; and evaluate performance consistency across image sources, generalization to unseen datasets, and the morphological relevance of model attributions.

**Methods:**

We evaluated binary normal-versus-abnormal sperm morphology classification using 7,770 microscopy images (1,253 normal and 6,517 abnormal sperm images) obtained from three independent institutional sources and representing abnormalities of the sperm head, neck/midpiece, and tail. Ten pretrained vision transformers and vision foundation-models spanning supervised, self-supervised, masked-image, and vision-language pretraining were evaluated using linear probing, light fine-tuning, and full fine-tuning. Five pretrained CNNs served as reference baselines. Primary analyses used source-stratified training, validation, and test partitions within each dataset to assess pooled performance, source-specific performance, and class-specific recall. Secondary analyses evaluated generalization to completely unseen datasets through leave-one-source-out validation and examined model attribution patterns using architecture-appropriate explainability methods.

**Results:**

SigLIP2 with full fine-tuning and ConvNeXt-Tiny achieved comparable weighted F1 scores (0.958 and 0.955, respectively), while BEiT with full fine-tuning and SigLIP2 with light fine-tuning achieved the highest ROC-AUC (0.982). SigLIP2 with full fine-tuning also produced the highest equal-weight cross-source macro-F1 (0.91) and normal-class recall (0.91). External generalization varied markedly across held-out image sources, with DINOv2 under full fine-tuning achieving ROC-AUCs ranging from 0.654 to 0.928 across the three external evaluations. Explainability analyses generally localized predictions to sperm-related structures.

**Conclusions:**

Pretraining strategy, adaptation depth, and dataset shift substantially influenced performance. Vision transformers and vision foundation models demonstrated strong potential for AI-assisted sperm morphology assessment, supporting their further development as scalable tools to improve the objectivity, consistency, and efficiency of semen analysis.

## 1 Introduction

Male infertility remains a major global health issue, and semen analysis continues to be central to its evaluation. Within this assessment, sperm morphology is particularly important because structural abnormalities of the head, neck, or tail may reflect impaired fertilizing capacity and broader reproductive dysfunction. However, morphology assessment is also one of the most variable components of semen analysis, as results are influenced by sample preparation, staining, image quality, microscope settings, and observer expertise. Even with computer-aided semen analysis systems, morphology remains challenging to standardize consistently in routine practice. These limitations have created strong motivation for artificial intelligence (AI)-assisted image analysis, which offers the possibility of more objective, scalable, and reproducible sperm evaluation in andrology laboratories (1–3).

AI-based sperm morphology analysis has evolved from conventional image-processing and machine-learning approaches to increasingly advanced deep-learning frameworks. Early studies demonstrated the feasibility of automated sperm morphology classification from microscopy images using conventional classifiers and CNN-based models, frequently improving upon handcrafted feature-engineering pipelines (4–7). Subsequent work introduced fusion-based and attention-enhanced architectures to further strengthen discrimination between normal and abnormal sperm forms (8–10). More recently, transformer-based models have also been investigated for sperm morphology analysis, reflecting the broader adoption of transformer architectures in biomedical imaging owing to their capacity to capture longer-range spatial dependencies and exploit richer pretrained visual representations (11).

Several studies have also adopted a more comparative or benchmark-like perspective. Yüzkat et al. developed six CNN models and evaluated hard- and soft-voting fusion across three public datasets, namely SMIDS, HuSHeM, and SCIAN-MorphoSpermGS, demonstrating the value of multi-model comparison in this domain (12). Spencer et al. then reported a stacked ensemble combining traditional and modern CNN architectures with a meta-classifier on HuSHeM and SCIAN (13). Shahzad et al. proposed a sequential deep neural network for the MHSMA benchmark and compared it against 11 pretrained deep-learning models (14). More recently, Aktas et al. presented a comparative ensemble framework on the Hi-LabSpermMorpho dataset (15) and, in a separate study, benchmarked eight vision transformer variants against CNN and hybrid baselines on SMIDS and HuSHeM (11).

More clinically oriented approaches have recently focused on the analysis of live, unstained sperm using whole-cell morphology. Shahali et al. developed an ensemble deep-learning model to classify live, unstained human sperm from whole-cell images, thereby avoiding staining procedures that render cells unsuitable for subsequent treatment. Yang et al. proposed a non-invasive multidimensional framework that combined multiple-sperm tracking with segmentation and compartment-level morphological analysis of the head, midpiece, and principal piece while simultaneously assessing motility. These approaches move beyond classification of retrospectively curated stained or cropped images and are more directly aligned with real-time sperm assessment and selection in assisted-reproduction workflows (16, 17).

Despite this progress, important gaps remain in the current evaluation literature. First, many prior studies are still dataset-specific, often using only one or two benchmarks, which limits insight into robustness across different staining conditions, acquisition settings, and labeling schemes (11, 13–15). Second, several widely used public datasets remain relatively small or morphologically narrow; for example, HuSHeM is focused on sperm head morphology, while other benchmarks provide restricted class diversity compared with the heterogeneity encountered in real laboratory practice (11, 14, 15). Third, recent transformer evaluations have largely focused on standard ViT variants rather than the broader ecosystem of modern pretrained vision encoders, such as self-supervised, masked-image-modeling, and vision-language models (11). Moreover, pooled performance alone may obscure substantial variation across image sources and does not establish whether models generalize to a completely unseen dataset. Taken together, these studies leave open a more practically important question: which pretrained visual representation families transfer best to sperm morphology analysis, how much task-specific adaptation is required, and how robustly do these models perform across heterogeneous and unseen image sources.

To address these gaps, we conducted a comparative benchmark of binary normal-versus-abnormal sperm morphology classification using three public datasets that capture variation in imaging characteristics and abnormalities of the head, neck/midpiece, and tail. Ten pretrained vision transformers and vision foundation-models spanning supervised, self-supervised, masked-image, and vision-language pretraining were evaluated under linear probing, light fine-tuning, and full fine-tuning, alongside five pretrained CNN baselines. Unlike clinically oriented systems designed for real-time analysis of live, unstained sperm, this study does not propose a deployable clinical platform. Rather, it provides a unified, source-aware methodological evaluation of pretrained visual representations, adaptation strategies, and performance consistency across heterogeneous microscopy datasets.

## 2 Methods

### 2.1 Study design and evaluation framework

**Figure 1:**
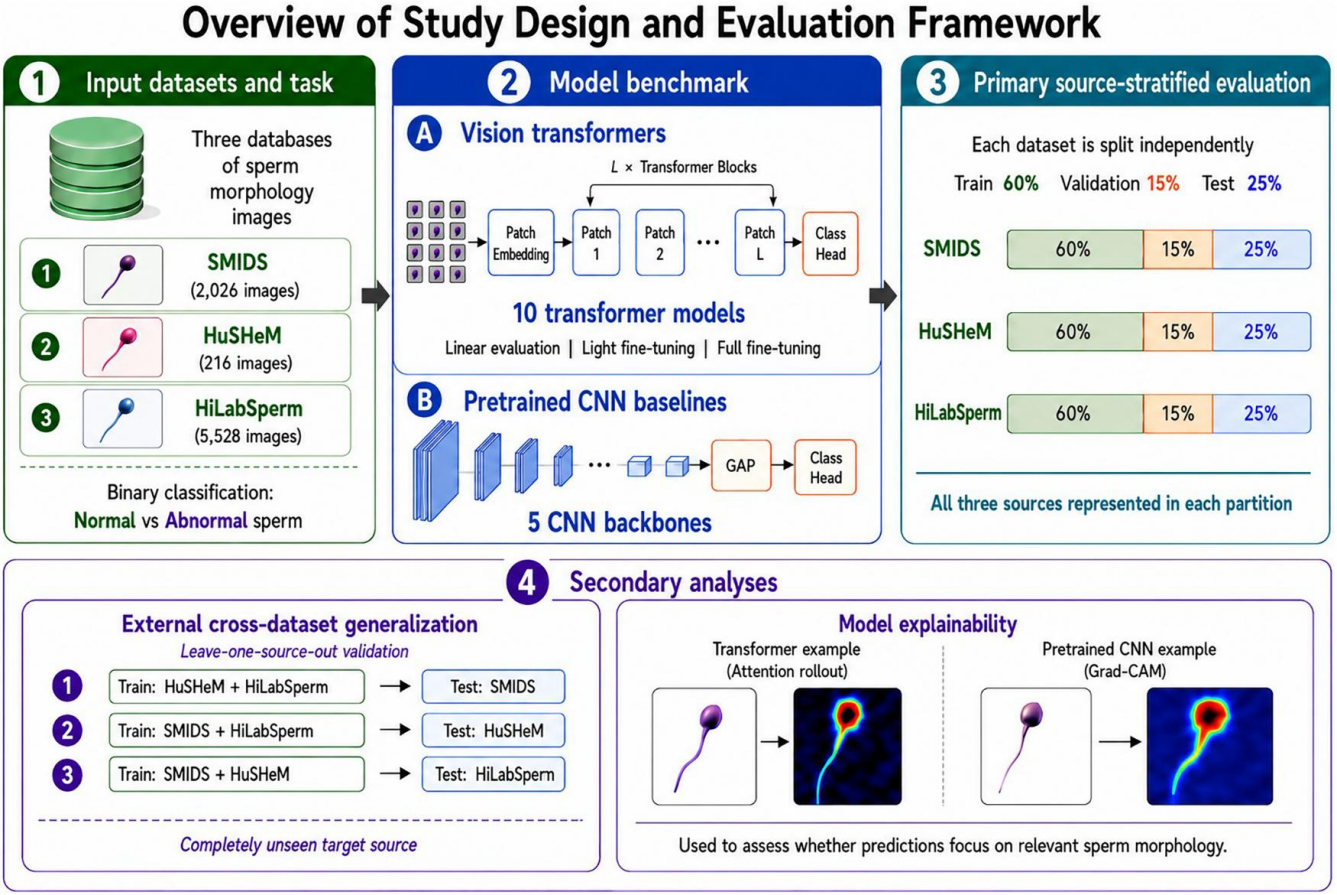
Overview of the study design and evaluation framework. Three public sperm morphology datasets were used to benchmark pretrained transformer models and pretrained CNN baselines under source-stratified evaluation. Primary analyses examined pooled and cross-source performance, while secondary analyses assessed external cross-dataset generalization and model explainability.

The study was designed as a multi-source benchmark comparing pretrained vision transformers and vision foundation-models with established CNN-based architectures for sperm morphology classification. Images from three public datasets were evaluated using a unified source-stratified framework in which each dataset contributed to the training, validation, and test partitions. The primary analyses assessed pooled discrimination and classification performance, consistency across image sources, and class-specific recall. Secondary analyses included leave-one-source-out validation to evaluate generalization to completely unseen datasets and architecture-appropriate explainability analyses using representative transformer- and CNN-based models. An overview of the complete study design and evaluation framework is presented in Figure 1.

### 2.2 Datasets

Three publicly available sperm morphology datasets were integrated to construct the benchmark used in this study: the Sperm Morphology Image Dataset (SMIDS) (18), the Human Sperm Head Morphology dataset (HuSHeM) (19), and HiLabSpermMorpho (20). Before model development, all images were screened during dataset assembly, and two extremely small images from HiLabSpermMorpho were excluded. The final merged benchmark comprised −7,770 microscopy images, including 1,253 normal sperm images and 6,517 abnormal sperm images (Table 1).

**Table 1:** Summary of the datasets used in this study.

Source dataset	Normal Sperm	Abnormal Sperm	Total
SMIDS	1,021	1,005	2,026
HuSHeM	54	162	216
HiLabSpermMorpho	178	5,350	5,528
Total	1,253	6,517	7,770

The Sperm Morphology Image Dataset (SMIDS) is a microscopy dataset derived from a smartphone-based data acquisition framework originally developed for semen analysis applications (4). The dataset contains 2,026 RGB images labeled as normal sperm (*n* = 1,021) and abnormal sperm (*n* = 1,005). A notable characteristic of SMIDS is that the abnormal category is provided as a single aggregate class rather than being subdivided into specific morphological subtypes. In addition, SMIDS contains variable image sizes and may include realistic acquisition artifacts such as noise and mixed tails, thereby reflecting a comparatively heterogeneous three-class classification scenario (18).

The Human Sperm Head Morphology dataset (HuSHeM) is focused specifically on head morphology. Samples were fixed and stained using the Diff-Quick method, imaged with an Olympus CX21 microscope using a ×100 objective and ×10 eyepiece, and captured with a Sony SSC-DC58AP color camera. From 725 microscopy images acquired at 576 × 720 pixels, sperm heads were cropped and classified by three specialists. Only samples with consensus labels were retained. The final HuSHeM dataset contains 216 RGB head crops of size 131 × 131 pixels distributed across four classes: normal (*n* = 54), tapered (*n* = 53), pyriform (*n* = 57), and amorphous (*n* = 52). In the present work, the three abnormal head subclasses were merged into a single abnormal sperm category, yielding 216 images in total when combined as 54 normal and 162 abnormal images. HuSHeM therefore contributed fine-grained head-specific abnormality examples but did not include neck, tail, or non-sperm categories (19).

HiLabSpermMorpho is a recently introduced expert-labeled sperm morphology dataset designed for abnormality detection and classification under multiple staining conditions. The dataset includes images prepared using Beslab HistoPlus and GBL staining methods and defines 18 morphological classes for each staining protocol. Unlike HuSHeM, HiLabSpermMorpho includes abnormalities affecting the sperm head, neck, and tail. In this study, we used the publicly available minimal subset and merged all abnormal subclasses into a single abnormal sperm category while retaining the normal class. After excluding two extremely small images, the dataset contributed 5,528 images, comprising 178 normal and 5,350 abnormal sperm images. The abnormal subclasses included amorphous, double, pyriform, round, tapered, pin, and vacuolated heads; narrow acrosomes; asymmetric, thick, thin, and twisted necks; and curly, double, long, short, and twisted tails. HiLabSpermMorpho therefore provided the largest and most morphologically diverse component of the merged benchmark (20).

**Figure 2:**
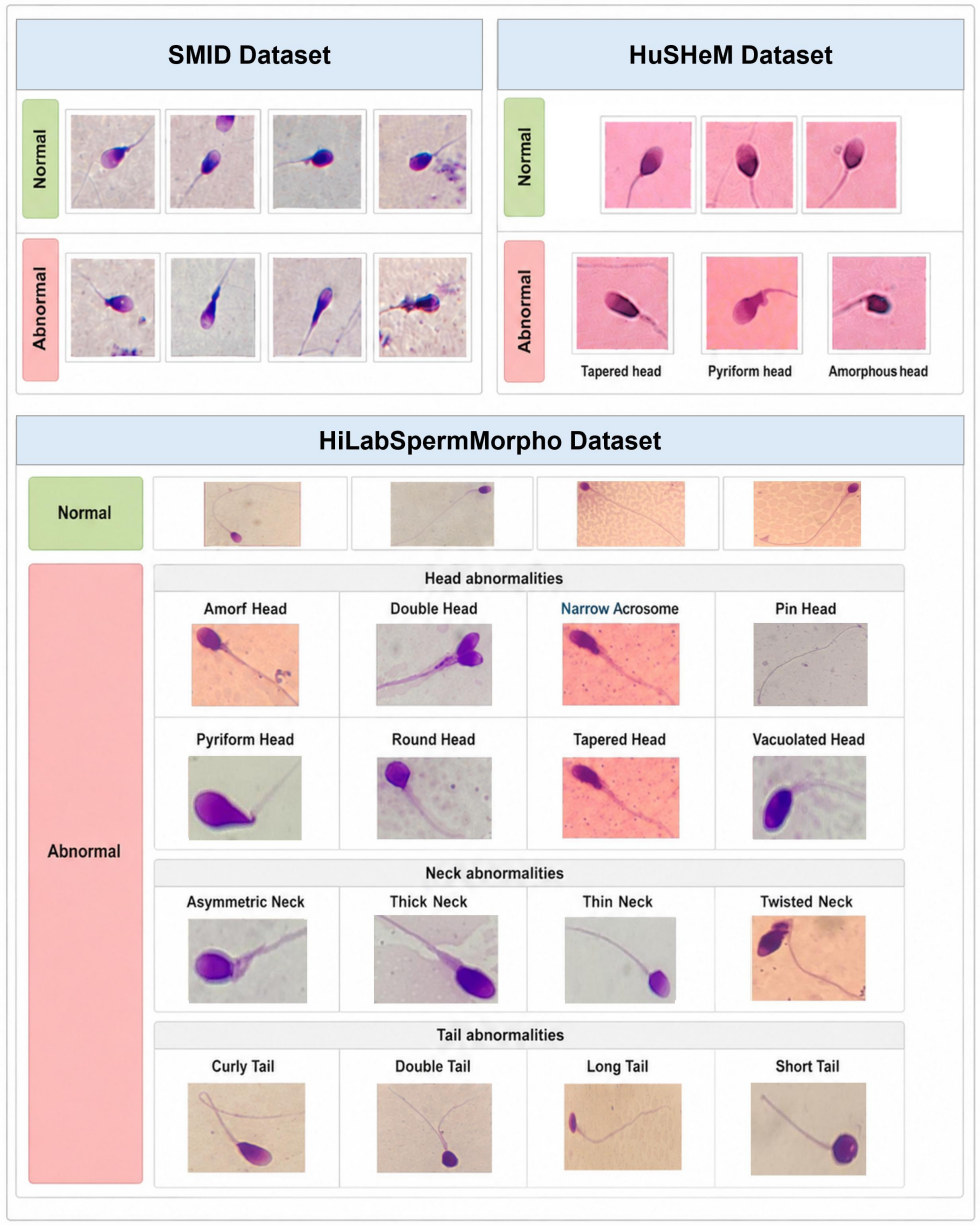
Representative normal and abnormal sperm morphology images from the three datasets included in this study. The upper-left section shows images from the SMID dataset, the upper-right section shows images from the HuSHeM dataset, and the lower section shows images from the HiLabSpermMorpho dataset.

Collectively, these three datasets capture complementary variation in image acquisition, staining characteristics, label granularity, and morphological abnormality coverage. Accordingly, the integrated benchmark enables evaluation in a realistic multi-source setting that combines coarse three-class discrimination, head-focused abnormal morphology, and broader fine-grained structural diversity across multiple sperm compartments. Representative examples from the three datasets are shown in Figure 2.

### 2.3 Transformer-based models and adaptation strategies

To evaluate transformer-based representation learning for sperm morphology classification, we constructed a benchmark spanning 10 pretrained model families selected to cover three complementary pretraining paradigms. The evaluated architectures were: (1) supervised image-only pretrained transformers: ViT-B/16, ViT-Small-PT, and DEiT; (2) self-supervised or masked-image-modeling transformers: DINOv2, DINOv2 with registers, ViT-MAE, and BEiT; and (3) vision-language pretrained models: CLIP, SigLIP2, and BiomedCLIP. This selection was designed to provide broad methodological coverage rather than focusing on a single transformer variant. Supervised pretrained vision transformers were included as strong and widely adopted reference architectures for image classification transfer. Self-supervised and masked-image-modeling families were incorporated to assess whether representation learning without explicit class supervision yields superior transferability for microscopy-based morphology analysis. Vision-language models were further included to examine whether multimodal pretraining confers additional benefit, particularly when image representations have been aligned with large-scale semantic supervision. Finally, BiomedCLIP was selected to complement the general-domain multimodal models by providing a domain-relevant biomedical vision-language baseline. Together, these model families enabled systematic comparison of diverse **vision transformer and vision foundation-model representations** in a unified binary sperm morphology classification benchmark.

For each family *f*, an input image *x_i_* was first transformed by the corresponding model-native preprocessing function $P_{f}(\cdot )$ and the resulting tensor was passed through the pretrained visual encoder $E_{f}(\cdot )$. A task-specific linear classification head with two output units was then attached to produce probabilities for the normal and abnormal sperm classes:

$$p_{i}=softmax(W_{f}z_{i}+b_{f}),$$

where $z_{i}$ denotes the latent visual representation extracted from the corresponding pretrained backbone, and $W_{f},b_{f}$ are the trainable classifier parameters**.** The output vector $p_{i}\in R^{2}$ contains the predicted probabilities for the normal and abnormal classes.

The exact form of $z_{i}$ depends on the model family. For ViT-B/16, implemented through the torchvision backbone, the final prediction head of the pretrained model was replaced by a linear layer with two output units, and classification relied on the encoder output of the original ViT architecture. For ViT-Small-PT, implemented with timm, the pretrained transformer was instantiated with num_classes=0 to expose feature embeddings, after which a separate linear classifier was applied; when the backbone output had spatial dimensions, global average pooling was used before classification. For the Hugging Face image-only transformer families: DINOv2, DINOv2 with registers, ViT-MAE, BEiT, and DEiT, the pretrained encoder was followed by a linear classifier operating primarily on the class-token representation $z_{i}=h_{i}^{\left[CLS\right]}$, extracted from the final hidden state. If a model exposed a pooled output rather than an explicit class token, the pooled representation was used instead. Thus, for these encoder-only families, prediction can be written as

$$p_{i}=softmax(W_{f}h_{i}^{\left[CLS\right]}+b_{f}),$$

or equivalently using the pooled encoder output when required by the model implementation.

The multimodal families were handled separately. For CLIP and SigLIP2, only the visual pathway was used for supervised classification. Specifically, the pretrained model-generated image embedding

$$z_{i}=g_{f}(P_{f}(x_{i}))$$

was obtained through the visual encoder and associated projection module, and a new linear classifier with two output units was optimized on top of this embedding:

$$p_{i}=softmax(W_{f}z_{i}+b_{f}).$$

BiomedCLIP was treated analogously using the OpenCLIP visual encoder, with the final projected image representation serving as input to the task-specific binary classification head.

Each model family was evaluated under three transfer-learning regimes: **linear probing**, **light fine-tuning**, and **full fine-tuning**. In the linear-probing setting, all pretrained backbone parameters were frozen and only the newly introduced classification head was optimized. This setting was used to assess the quality of the pretrained visual representations when transferred to sperm morphology classification without modifying the underlying encoder. In the light fine-tuning setting, the classification head remained trainable and the final two transformer blocks were additionally unfrozen, together with associated normalization layers and, when applicable, visual projection parameters. This provided a controlled form of adaptation in which only the upper portion of the pretrained model was updated. In the full fine-tuning setting, all model parameters were optimized end-to-end, allowing the entire network to adapt to the target task.

Operationally, the exact implementation of light fine-tuning was tailored to each architecture while preserving the same general principle of limited upper-layer adaptation. For torchvision ViT-B/16, the final two encoder blocks and encoder normalization were unfrozen. For timm ViT-Small-PT, the final two transformer blocks and final normalization layer were updated. For the Hugging Face image-only transformer families, namely DINOv2, DINOv2 with registers, ViT-MAE, BEiT, and DEiT, the final two encoder layers together with available layer-normalization modules were unfrozen. For CLIP and SigLIP2, the final two visual encoder blocks, along with visual projection and post-layer-normalization components when available, were updated. For BiomedCLIP, the last two visual transformer residual blocks, post-transformer normalization, and visual projection parameters were unfrozen. This design enabled controlled comparison of fixed-feature transfer, partial adaptation, and full end-to-end adaptation across diverse transformer and foundation-model families.

Training used class-weighted cross-entropy with label smoothing. For a training sample iwith binary label $y_{i}\in \{1,2\}$, representing the normal and abnormal classes, predicted probabilities $p_{ic}$ for class *c*, class weight $w_{y_{i}}$, number of classes K=2**,** and smoothing factor ε=0.05, the loss was

$$L=-\frac{1}{N}\overset{N}{\underset{i=1}{\sum }}\;w_{y_{i}}\overset{2}{\underset{c=1}{\sum }}\;{\tilde{y}}_{ic}log(p_{ic}),$$

where the label-smoothed binary target was defined as

$${\tilde{y}}_{ic}=\{\begin{array}{cc} 1-\varepsilon +\frac{\varepsilon }{2}, & c=y_{i},\\ \frac{\varepsilon }{2}, & c\neq y_{i}. \end{array}$$

Optimization was performed with AdamW and cosine-annealing learning-rate scheduling using a batch size of 64. Linear probing used 12 epochs with head learning rate $10^{-3}$; light fine-tuning used 12 epochs with head learning rate 5 × 10^−4^ and backbone learning rate 2 × 10^−5^; and full fine-tuning used 10 epochs with head learning rate 3 × 10^−4^ and backbone learning rate 10^−5^. Weight decay was fixed at 10^−4^. The best checkpoint for each experiment was selected according to validation F1-score.

### 2.4 Pretrained CNN-backbone baselines

To provide strong convolutional reference models for comparison with the transformer-based benchmark, five ImageNet-pretrained CNN backbones were evaluated: ResNet50, DenseNet121, EfficientNetV2-M, MobileNetV2, and ConvNeXt-Tiny. These architectures were selected to represent complementary convolutional design paradigms, including residual learning, dense feature reuse, efficiency-oriented scaling, and modernized convolutional design.

For each model, the pretrained feature extractor was retained and the original classifier was replaced with a new linear layer containing two output units for binary normal-versus-abnormal sperm classification. Inputs were processed through a unified preprocessing pipeline and then adapted to the input size required by the pretrained backbone. All pretrained CNN baselines were optimized using supervised fine-tuning with differential learning rates, assigning a smaller learning rate to the pretrained backbone and a larger learning rate to the newly initialized classification head to promote stable transfer while allowing rapid task-specific adaptation.

Training employed class-weighted cross-entropy loss with label smoothing, AdamW optimization, and cosine-annealing learning-rate scheduling. ResNet50, DenseNet121, and EfficientNetV2-M were trained for 20 epochs, whereas ConvNeXt-Tiny was trained for 18 epochs. Model selection was based exclusively on validation performance, and the best checkpoint for each backbone was retained for final testing.

### 2.5 Preprocessing

For the transformer/foundation models and the pretrained CNN-backbone baselines, image preprocessing was kept minimal and limited to model-compatible operations required to match the expected input format of each pretrained architecture. For the vision transformers and vision foundation-models, native preprocessing pipelines associated with each pretrained model were used, including resizing, scaling, tensor conversion, and normalization. For the pretrained CNN-backbone models, a unified preprocessing pipeline was applied, consisting of resizing and center cropping, tensor conversion, and ImageNet-compatible normalization. All models received images at an effective input resolution of 224 × 224 pixels. Training-time augmentation, where applied, was restricted to the training partitions, whereas validation and test images underwent deterministic preprocessing without augmentation.

### 2.6 Architecture-appropriate model explainability

To assess whether predictions were driven by relevant sperm morphology rather than background or dataset-specific artefacts, architecture-appropriate attribution methods were applied to one representative transformer model and one representative CNN-based model. Representative examples from each source dataset included normal, abnormal, and misclassified cases where available.

For the transformer model, class-contrastive gradient-weighted attention rollout was used. Layer-wise attention matrices were weighted by gradients of the margin between the predicted and alternative classes, averaged across heads, combined with residual connections, and propagated across layers. Spatial patch scores were then reshaped and upsampled to the input resolution. For the CNN-based model, class-contrastive Grad-CAM was applied to the final convolutional block. Gradient-derived channel weights were combined with activation maps, rectified, normalized, and resized to the original image dimensions. Attribution maps were displayed with the model inputs and as heatmap overlays, with warmer colors indicating stronger contributions to the prediction.

### 2.7 Evaluation setup

The primary benchmark comprised 35 model configurations: 30 transformer-based configurations derived from 10 architectures evaluated under three adaptation strategies—linear probing, light fine-tuning, and full fine-tuning—and five pretrained CNN-backbone baselines. All models addressed the same binary image-level classification task: normal versus abnormal sperm. Each source dataset was independently divided into training (60%), validation (15%), and test (25%) partitions using class-stratified sampling. The corresponding source-specific partitions were subsequently combined, ensuring that all three datasets were represented in each partition while preserving their within-source class distributions. Class imbalance was addressed using class-weighted cross-entropy loss with label smoothing. Model selection and any decision-threshold optimization were performed exclusively on the validation data, after which the selected checkpoint and threshold were fixed for test-set evaluation.

Train, validation, and test partitioning was performed at the image level independently within each source dataset, with each indexed image assigned to only one partition. Consistent patient-, specimen-, slide-, or acquisition-level identifiers were not available across the public datasets; therefore, group-wise separation of visually related images originating from the same acquisition context could not be implemented. To address broader source-related overlap, we additionally performed leave-one-source-out validation in which the complete target dataset was excluded from model development.

The primary analyses evaluated pooled discrimination using receiver operating characteristic area under the curve (ROC-AUC) and pooled classification performance using weighted F1. Source-specific macro-F1 and the equal-weight mean across the three sources were used to assess performance consistency without allowing the largest dataset to dominate the summary estimate. Recall was also examined separately for the normal and abnormal classes, with each source contributing equally to the class-specific summaries.

Secondary analyses included leave-one-source-out validation, in which each dataset was evaluated as a completely unseen external source without target-source training, validation, threshold selection, or adaptation. Bootstrap 95% confidence intervals were calculated for transformer-model estimates, using source-stratified resampling for the multi-source test set. For CNN baselines, 95% confidence intervals were calculated across the five independent seeds. All experiments were conducted on an NVIDIA A100 80 GB GPU.

## 3 Results

### 3.1 Analysis overview

The analyses were structured to address five complementary questions. First, the primary benchmark used source-stratified 60%/15%/25% training, validation, and test splits within SMIDS, HuSHeM, and HiLabSpermMorpho, ensuring that all three sources were represented in each partition. Pooled AUC and weighted F1 were used to compare the overall performance of vision transformer configurations with pretrained CNN baselines. Second, source-specific and equal-weight Macro-F1 were examined to determine whether performance was consistent across datasets rather than driven by differences in source size. Third, normal- and abnormal-class recall were evaluated separately to assess class-specific performance. Fourth, leave-one-source-out validation tested generalization when each dataset was treated as a completely unseen external source.

Finally, architecture-appropriate explainability analyses were performed on representative transformer- and CNN-based models to assess whether model predictions focused on relevant sperm morphology.

### 3.2 Overall discrimination and pooled classification performance

**Figure 3:**
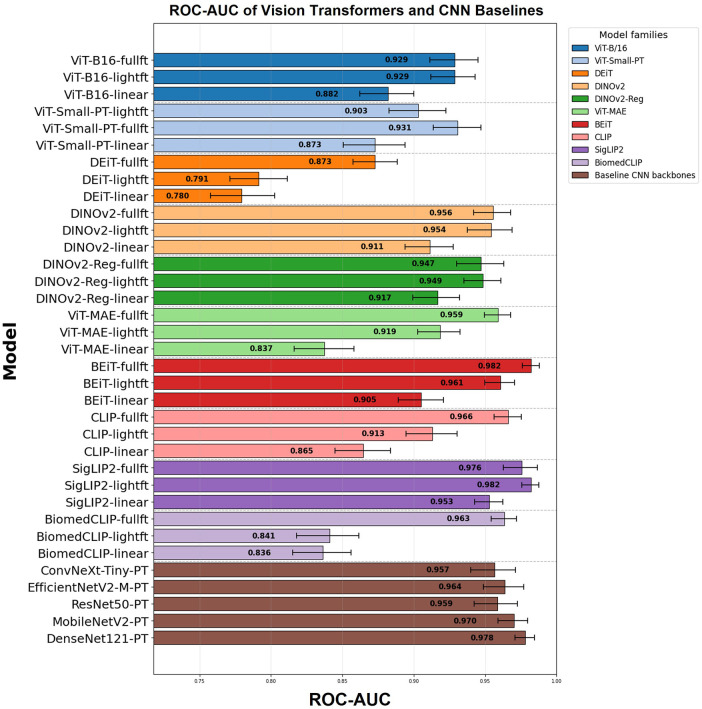
ROC-AUC of vision transformers and CNN baselines. Error bars show bootstrap 95% confidence intervals for transformer models and 95% confidence intervals across five seeds for CNN baselines.

Overall pooled ROC-AUC was used as a threshold-independent measure of discrimination between normal and abnormal sperm images (Figure 3). Among the vision transformer models, BEiT with full fine-tuning and SigLIP2 with light fine-tuning achieved the highest ROC-AUC values, both at 0.982. The strongest CNN baseline, DenseNet121-PT, achieved an ROC-AUC of 0.978, followed by SigLIP2 with full fine-tuning at 0.976 and MobileNetV2-PT at 0.970. Within several transformer families, full fine-tuning yielded higher ROC-AUC than linear evaluation, including BEiT (0.905 vs. 0.982), CLIP (0.865 vs. 0.966), and ViT-MAE (0.837 vs. 0.959). Source-stratified bootstrap 95% confidence intervals are shown as error bars.

**Figure 4:**
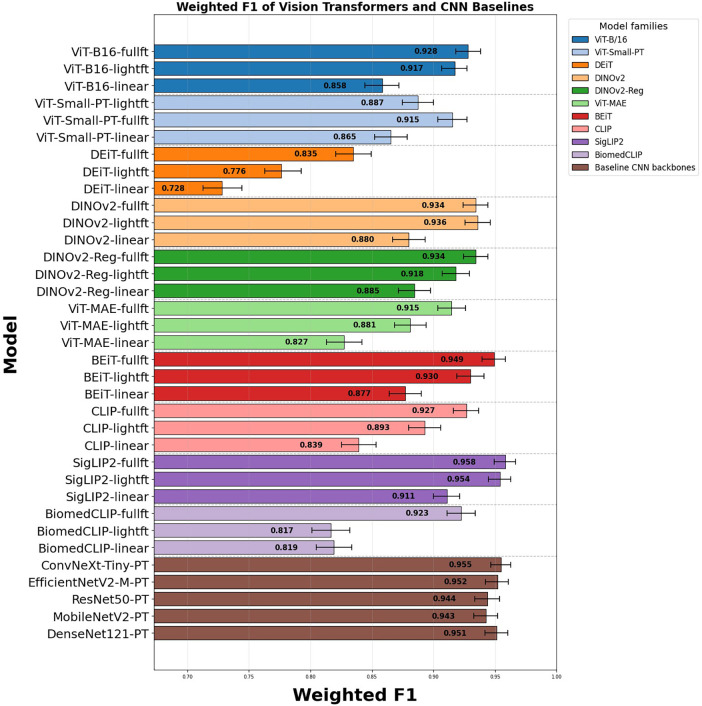
Weighted F1 of vision transformers and CNN baselines. Error bars show bootstrap 95% confidence intervals for transformer models and 95% confidence intervals across five seeds for CNN baselines.

Weighted F1 was used to summarize pooled classification performance while accounting for class support (Figure 4). SigLIP2 with full fine-tuning achieved the highest weighted F1 (0.958), narrowly exceeding ConvNeXt-Tiny-PT (0.955), SigLIP2 with light fine-tuning (0.954), EfficientNetV2-M-PT (0.952), and DenseNet121-PT (0.951). BEiT with full fine-tuning also achieved a weighted F1 of 0.949. Full fine-tuning produced higher weighted F1 than linear evaluation within several transformer families, including ViT-B/16 (0.858 vs. 0.928), DINOv2 (0.880 vs. 0.934), BEiT (0.877 vs. 0.949), and CLIP (0.839 vs. 0.927). Detailed pooled confusion metrics for the transformer models are provided in Supplementary Figure S1A,B.

### 3.3 Consistency of performance across image sources

**Figure 5:**
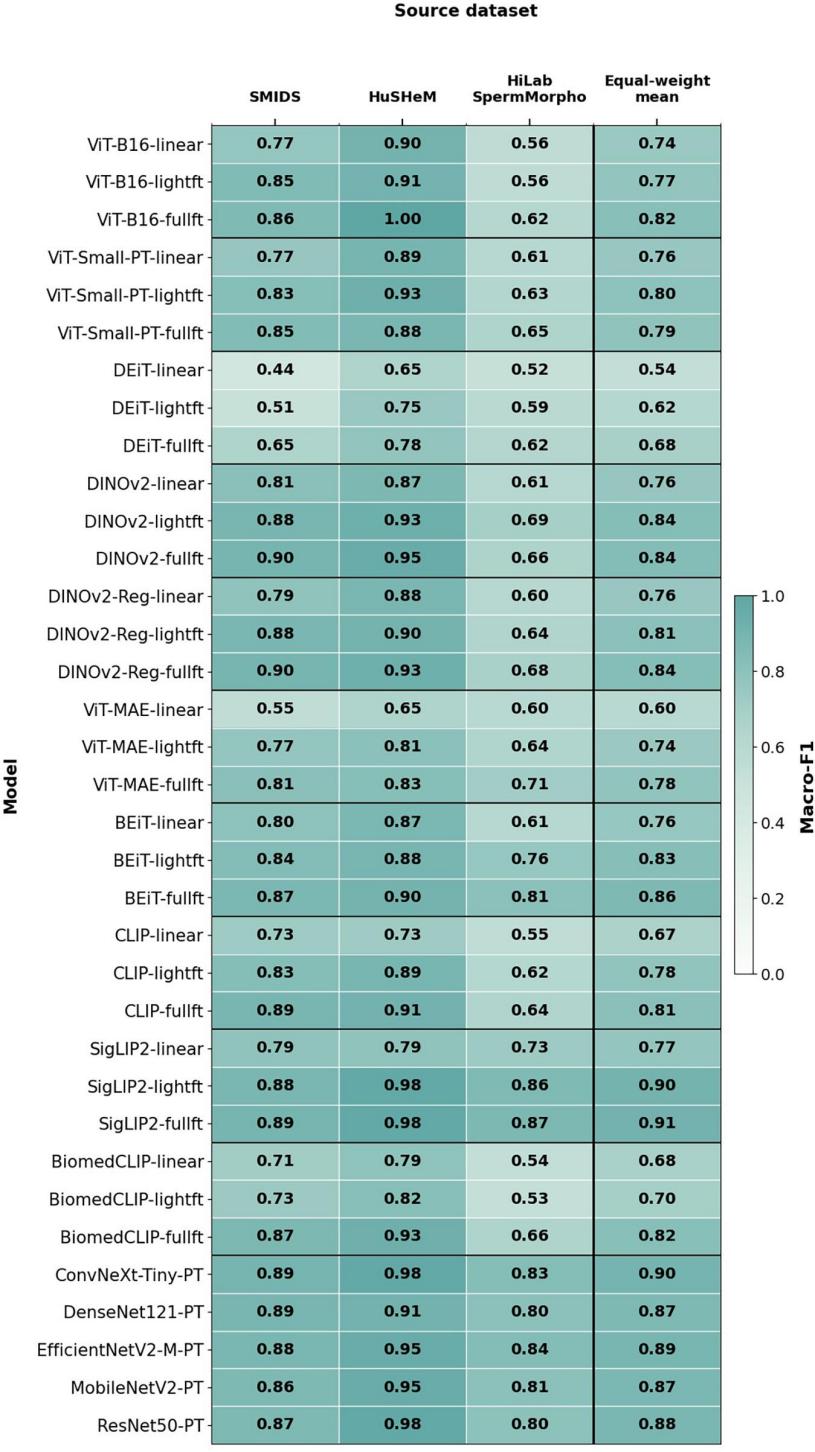
Cross-source macro-F1 performance across the evaluated vision transformer models and pretrained CNN baselines.

Because the three datasets differed substantially in size, an equal-weight mean was used so that SMIDS, HuSHeM, and HiLabSpermMorpho contributed equally to the cross-source summary rather than allowing the largest dataset to dominate the result. Source-specific and equal-weight Macro-F1 values were examined to assess whether model performance was consistent across the three image sources (Figure 5). SigLIP2 with full fine-tuning achieved the highest equal-weight mean Macro-F1 (0.91), with values of 0.89 for SMIDS, 0.98 for HuSHeM, and 0.87 for HiLabSpermMorpho. SigLIP2 with light fine-tuning and the CNN baseline ConvNeXt-Tiny-PT both achieved an equal-weight mean of 0.90. EfficientNetV2-M-PT followed with 0.89, while ResNet50-PT achieved 0.88.

Performance differed across image sources. HuSHeM generally yielded the highest Macro-F1 values, whereas HiLabSpermMorpho produced lower values for many models. For example, ViT-B/16 with full fine-tuning achieved a Macro-F1 of 1.00 on HuSHeM and 0.62 on HiLabSpermMorpho, resulting in an equal-weight mean of 0.82. SigLIP2 and the leading CNN baselines showed higher Macro-F1 values across all three datasets.

### 3.4 Normal- and abnormal-class recall

**Figure 6:**
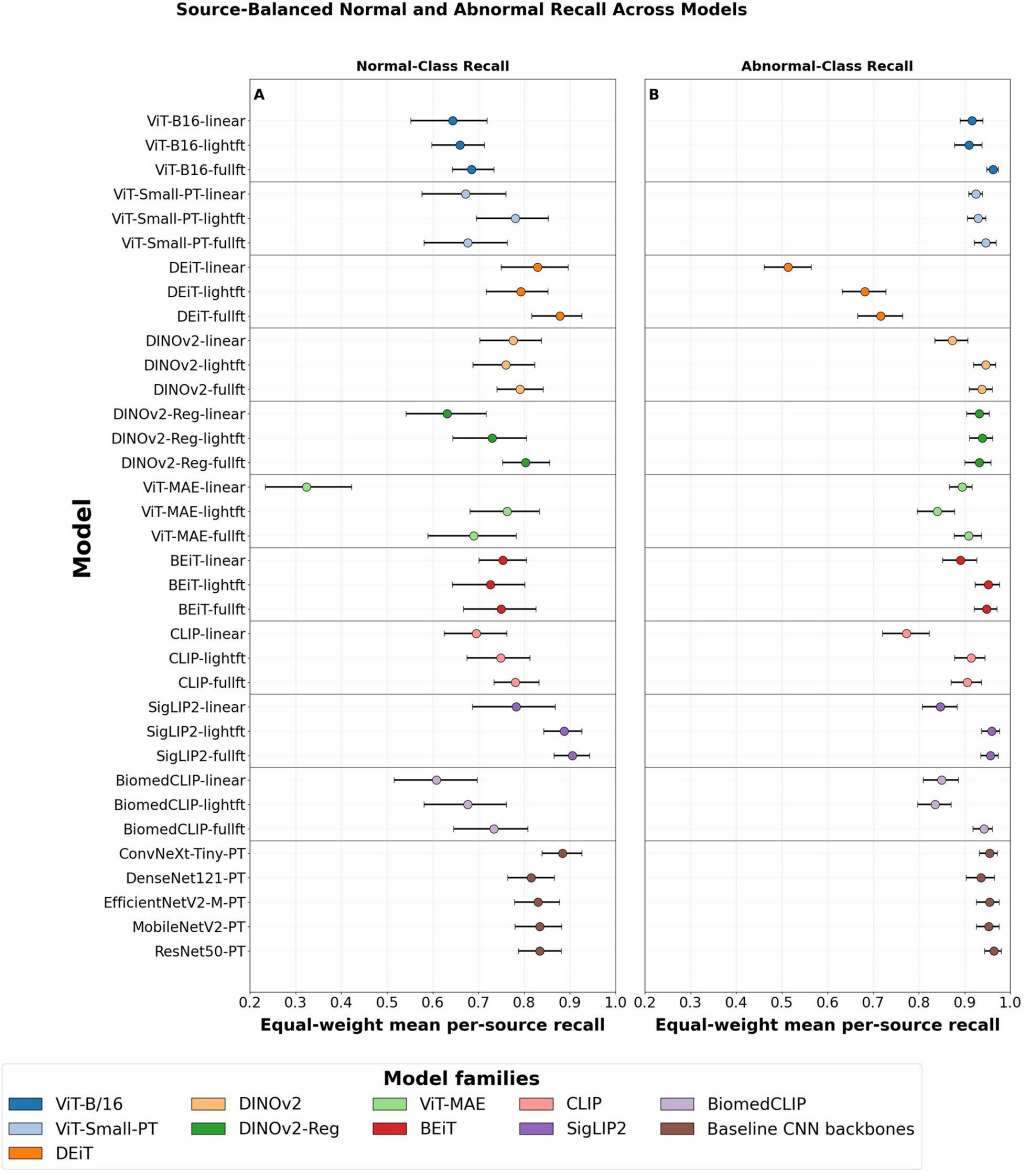
Source-Balanced normal- and abnormal-class recall across the evaluated vision transformer models and pretrained CNN baselines.

Equal-weight recall was examined separately for normal and abnormal sperm images to assess class-specific performance while giving each dataset equal contribution (Figure 6). Abnormal-class recall was numerically higher than normal-class recall for most models, although the difference varied across transformer architectures, fine-tuning strategies, and CNN baselines.

SigLIP2 with full fine-tuning achieved the highest normal-class recall (0.91), together with an abnormal-class recall of 0.96. SigLIP2 with light fine-tuning achieved corresponding values of 0.89 and 0.96. Among the CNN baselines, ConvNeXt-Tiny-PT achieved normal- and abnormal-class recalls of 0.88 and 0.95, respectively. The remaining CNN models achieved normal-class recall values of 0.82–0.83 and abnormal-class recall values of 0.94–0.96.

Some transformer configurations showed larger differences between the two classes. ViT-MAE under linear evaluation achieved a normal-class recall of 0.32 and an abnormal-class recall of 0.89. DEiT under linear evaluation achieved normal- and abnormal-class recalls of 0.83 and 0.51, respectively, while full fine-tuning increased the corresponding values to 0.88 and 0.72. Source-stratified bootstrap 95% confidence intervals are shown for the reported estimates.

### 3.5 External cross-dataset generalization in leave-one-source-out validation

Leave-one-source-out validation was performed to assess generalization to a completely unseen image source. For each experiment, models were trained and validated using two datasets and evaluated on the third dataset without target-source training, validation, or adaptation. Specifically, models were trained on HuSHeM and HiLabSpermMorpho when SMIDS was held out, on SMIDS and HiLabSpermMorpho when HuSHeM was held out, and on SMIDS and HuSHeM when HiLabSpermMorpho was held out, respectively (Supplementary Figures S2A–C).

When SMIDS was held out, DINOv2 with full fine-tuning achieved the highest ROC-AUC (0.844), followed by DINOv2 under linear evaluation (0.828) and DINOv2-Reg with light fine-tuning (0.810). The highest-performing CNN was EfficientNetV2-M-PT, with an AUC of 0.580 (Supplementary Figure S2A). When HuSHeM was held out, DINOv2 with full fine-tuning again achieved the highest AUC (0.928), followed by BEiT under linear evaluation (0.916), BEiT with light fine-tuning (0.910), and ViT-Small-PT with light fine-tuning (0.899). DenseNet121-PT was the strongest CNN in this setting, with an AUC of 0.802 (Supplementary Figure S2B).

Performance was substantially lower when HiLabSpermMorpho was held out. DINOv2 with full fine-tuning achieved the highest AUC (0.654), followed by ViT-B/16 under linear evaluation (0.629), DINOv2-Reg with full fine-tuning (0.579), and SigLIP2 with full fine-tuning (0.576). ResNet50-PT was the highest-performing CNN, with an AUC of 0.570 (Supplementary Figure S2C). Overall, cross-dataset generalization was strongly dependent on the held-out source, with HuSHeM producing the highest external-validation performance and HiLabSpermMorpho representing the most challenging unseen domain. Error bars show bootstrap 95% confidence intervals for transformer models and 95% confidence intervals across five seeds for CNN baselines.

### 3.6 Model explainability using attention rollout and Grad-CAM

**Figure 7:**
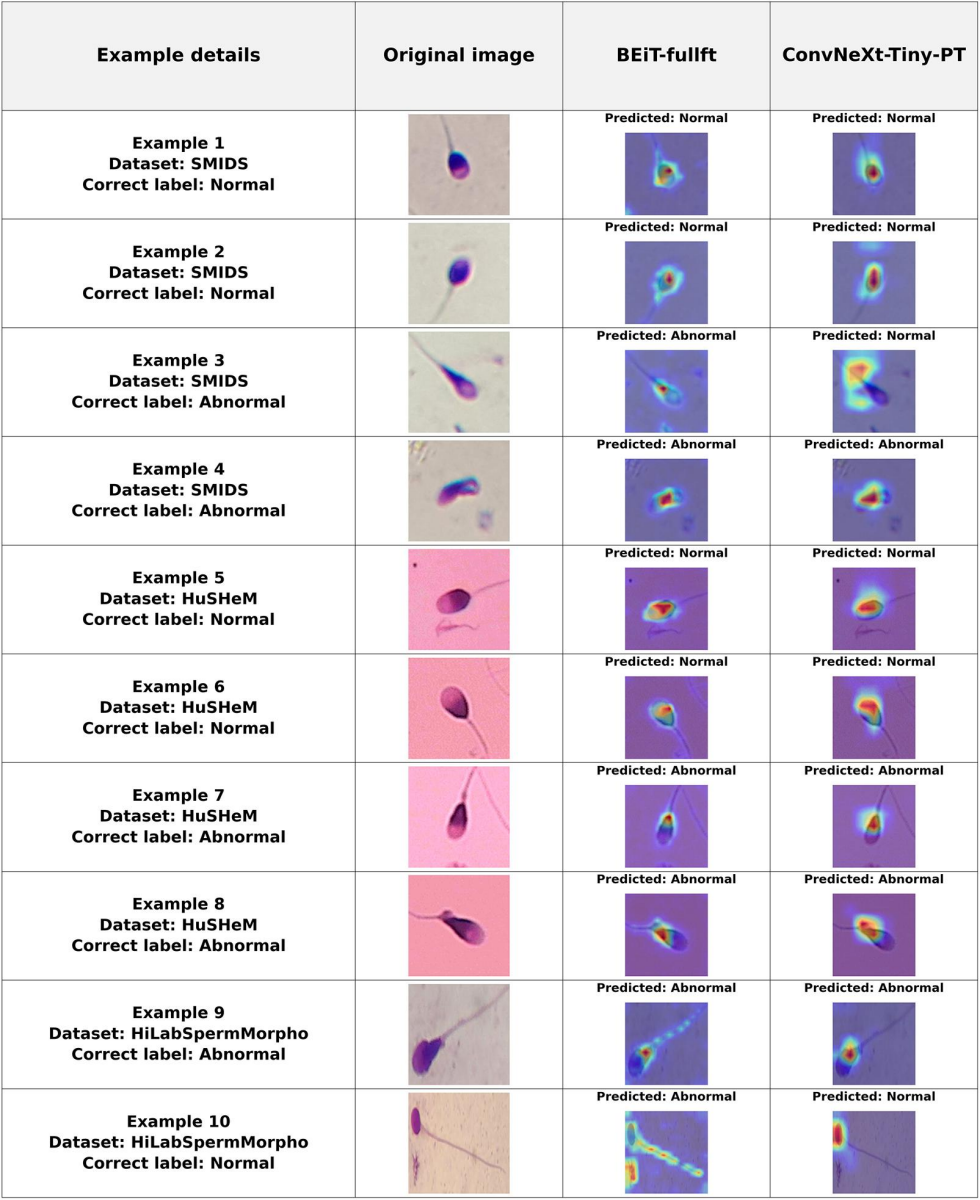
Comparative explainability examples for representative transformer- and CNN-based models. Ten test images from the three source datasets are shown with their correct labels, model predictions, and attribution overlays. Transformer explanations were generated using gradient-weighted attention rollout, while CNN explanations used Grad-CAM. Warmer colors indicate regions contributing more strongly to each prediction.

For the explainability analysis, one representative transformer model, BEiT-fullft, and one representative CNN-based model, ConvNeXt-Tiny-PT, were selected. Both models generally assigned their strongest attribution to sperm-related structures, particularly the head and adjacent midpiece regions (Figure 7). In several correctly classified examples, the models produced broadly similar localization patterns, although the extent and distribution of highlighted regions differed. Some cases also showed attribution extending along the tail or into surrounding image regions, particularly for elongated structures or complex backgrounds. Differences between model predictions were observed in selected cases; for example, the models disagreed on Examples 3 and 10. These findings indicate that similar predictions may arise from partially different spatial evidence. Additional transformer explainability examples are provided in Supplementary Figure S3, while Supplementary Figure S4 presents examples for CNN-based models.

## 4 Discussion

### 4.1 Main findings

This study provides a broad evaluation of pretrained vision transformers, vision foundation models, and CNN backbones for binary normal-versus-abnormal sperm morphology classification using a heterogeneous multi-source microscopy benchmark. Several important findings emerged.

First, adaptation strategy substantially influenced performance, although its effect was not uniform across all model families or evaluation settings. Full fine-tuning generally improved pooled classification performance relative to linear probing and frequently produced the strongest results within individual transformer families. However, light fine-tuning remained highly competitive for some architectures; notably, SigLIP2 with light fine-tuning achieved one of the highest pooled ROC-AUC values. These findings indicate that sperm morphology representations often benefit from task-specific adaptation, but complete end-to-end optimization is not invariably required to achieve strong discrimination.

Second, no single architecture or pretraining paradigm was uniformly superior across all outcomes. SigLIP2 with full fine-tuning achieved the highest pooled weighted F1 and the highest equal-weight cross-source macro-F1, whereas BEiT with full fine-tuning and SigLIP2 with light fine-tuning achieved the highest pooled ROC-AUC. DINOv2 with full fine-tuning performed particularly strongly in leave-one-source-out validation. Collectively, these results suggest that supervised, self-supervised, masked-image, and vision-language pretraining can all provide useful representations for sperm morphology analysis, but their relative advantages depend on the performance measure and evaluation context.

Third, the pretrained CNN baselines remained highly competitive. ConvNeXt-Tiny achieved pooled classification performance close to that of the strongest transformer configurations, while DenseNet121 produced ROC-AUC comparable to the leading transformer models. The findings therefore do not support a universal advantage of transformer-based models over convolutional architectures. Instead, they demonstrate that both architecture families can provide strong performance when pretrained and appropriately adapted, reinforcing the importance of comparison against robust CNN baselines.

Fourth, performance varied meaningfully across image sources and classes. Source-balanced analyses showed that high pooled performance did not necessarily imply equally strong performance across SMIDS, HuSHeM, and HiLabSpermMorpho. Abnormal-class recall was also higher than normal-class recall for most models, indicating that aggregate metrics can obscure clinically relevant differences between the two classes. These results underscore the value of source-specific and class-specific reporting, particularly in benchmarks containing substantial differences in dataset size, staining, image acquisition, cropping, and abnormality coverage.

Finally, leave-one-source-out validation revealed a marked reduction and substantial variability in performance when models were applied to completely unseen datasets. Generalization was strongest when HuSHeM was held out and weakest when HiLabSpermMorpho was treated as the external source. This pattern likely reflects differences in image characteristics, annotation scope, and morphological diversity across the datasets and demonstrates that strong performance when all sources are represented during development should not be interpreted as evidence of robust cross-dataset generalization. Explainability analyses provided complementary evidence that both representative transformer and CNN models generally focused on sperm-related structures, particularly the head and adjacent midpiece, although attribution occasionally extended to surrounding regions or image background.

### 4.2 Research and clinical implications

From a research perspective, the findings demonstrate that the evaluation of pretrained visual models for sperm morphology should not rely solely on pooled performance from a combined multi-source test set. Although pooled ROC-AUC and weighted F1 provide useful summaries of discrimination and overall classification performance, they can conceal substantial variation across image sources, particularly when datasets differ markedly in size, staining protocol, image scale, cropping, acquisition hardware, background appearance, and abnormality coverage. In the present study, source-specific macro-F1 and class-specific recall revealed performance differences that were not fully apparent from pooled metrics. This indicates that future sperm morphology benchmarks should report source-level results alongside aggregate performance and should use source-balanced summaries when one dataset contributes a disproportionate share of the test images.

The leave-one-source-out experiments further show that performance obtained when all datasets are represented during model development should not be interpreted as evidence of external generalizability. The marked variation across held-out datasets, and particularly the lower performance when HiLabSpermMorpho was treated as an unseen source, suggests that cross-dataset transfer depends on more than the distinction between normal and abnormal morphology. Differences in staining, field composition, sperm compartment coverage, annotation scope, and image acquisition may alter the visual representation of the same biological class. Consequently, source identity may become entangled with class-relevant features, allowing models to perform well within a familiar multi-source distribution while remaining less reliable in a genuinely new laboratory domain. This has important implications for reproductive-imaging research: source-stratified evaluation and leave-one-source-out testing should be viewed as complementary rather than interchangeable, with the former measuring performance within a heterogeneous but represented distribution and the latter providing a more stringent test of domain transfer.

The results also provide insight into the role of pretraining and downstream adaptation. The generally weaker performance of linear probing indicates that the normal-versus-abnormal distinction is not consistently separable using fixed off-the-shelf embeddings. Fine-tuning appears to be important for aligning pretrained representations with the subtle structural and textural characteristics of sperm microscopy. However, the strong performance of selected light fine-tuning configurations shows that full end-to-end optimization is not always necessary. This is particularly relevant for smaller biomedical datasets, where unrestricted fine-tuning may increase computational cost and the risk of overfitting. The practical research question is therefore not simply which architecture performs best, but how much adaptation is required for a given pretrained representation to become useful in a morphology-sensitive and source-heterogeneous setting.

The competitive performance of the strongest CNN baselines has an additional methodological implication. It shows that vision transformers and vision foundation models should not be assumed to provide an automatic advantage over modern convolutional architectures. Their value may instead lie in specific properties such as transfer flexibility, representation richness, compatibility with self-supervised or multimodal pretraining, and potential reuse across related tasks. Future studies should therefore evaluate not only discrimination, but calibration, uncertainty, computational efficiency, source robustness, and interpretability. Such comparisons would provide a more meaningful basis for selecting models for reproductive imaging than rankings based on a single pooled metric.

Clinically, the class-specific results are important because the consequences of error are not symmetric. Lower normal-class recall in several configurations indicates a tendency to label some normal sperm as abnormal, even when aggregate performance remains high. In practice, such behavior could increase apparent abnormality rates, create unnecessary review burden, or reduce confidence in automated support. Conversely, failure to identify abnormal morphology could provide false reassurance. Clinical translation will therefore require explicit evaluation of class-specific sensitivity, operating thresholds, probability calibration, and uncertainty. Rather than producing an isolated categorical label, a clinically useful system should ideally provide a confidence estimate, flag borderline cases, and direct uncertain images to expert review.

Within routine practice, sperm morphology is interpreted as part of the broader WHO-aligned semen analysis framework (21) rather than as an isolated image-classification task. Accordingly, the present models should be viewed as decision-support components that may assist morphology assessment within standardized laboratory workflows rather than replace complete clinical semen reporting. Their near-term value may lie in supporting pre-screening, second reading, quality assurance, and consistency across operators. For example, a model could prioritize images for review, identify cases with low confidence, or provide visual attribution maps to indicate the structures contributing to a prediction. However, these functions would need to be validated prospectively using locally acquired images and compared with expert interpretation under routine laboratory conditions.

The explainability findings also have practical implications. The tendency of both the representative transformer and CNN models to focus on sperm-related structures, particularly the head and adjacent midpiece, is encouraging because these regions contain clinically relevant morphological information. Nevertheless, attribution extending into surrounding regions in some images indicates that models may still use contextual or source-specific visual cues. Explainability should therefore be used as a quality-control and hypothesis-generation tool rather than as proof of clinically valid reasoning. Future work could strengthen interpretability by combining classification with explicit segmentation or localization of the head, neck/midpiece, and tail, allowing model predictions to be linked more directly to defined morphological compartments.

Finally, these findings suggest that AI systems for sperm morphology should be viewed not merely as classification tools, but as components of a broader digital andrology workflow. When combined with automated sperm detection, image-quality control, segmentation, and future integration with motility or concentration measurements, morphology classifiers could contribute to more comprehensive and reproducible computer-aided semen analysis pipelines. The pretrained encoders evaluated in this study may also provide reusable representations for related tasks, including abnormality localization, subtype classification, quality assessment, and multimodal integration with laboratory and clinical data. The present results therefore have implications beyond model benchmarking: they support the feasibility of developing assistive systems that improve consistency in morphology assessment while creating a foundation for richer multimodal fertility AI in the future.

### 4.3 Limitations and future work

A main limitation is that all abnormalities were combined into a single class, although individual sperm may exhibit concurrent defects across the head, neck/midpiece, and tail. This was dictated by inconsistent subclass definitions and the absence of harmonized multi-label annotations across the utilized public datasets. In addition, specimen- or acquisition-level identifiers were unavailable, so visually related images may have been distributed across the primary partitions, although leave-one-source-out validation removed dataset-level overlap. Future studies should use harmonized multi-label annotations and patient-, slide-, or acquisition-grouped splitting when such metadata are available.

A second limitation is the substantial heterogeneity among the three datasets in staining protocols, acquisition conditions, image scale, class distribution, annotation granularity, and abnormality coverage. Although this diversity enabled source-specific and leave-one-source-out evaluation, it may also have introduced source-related visual cues and influenced learned decision boundaries. Moreover, the markedly different dataset sizes meant that some morphological patterns were represented more extensively than others. Future studies should therefore prioritize larger, prospectively collected, multi-center datasets with standardized annotations and should evaluate generalization across unseen laboratories, microscopes, staining methods, and patient populations.

Finally, the study was retrospective and limited to image-level classification. It did not assess probability calibration, agreement with expert readers, clinical operating thresholds, or effects on laboratory efficiency and reporting. The explainability analysis was also *post hoc* and restricted to representative models, and therefore cannot establish that model decisions reflect clinically valid biological reasoning. Future research should incorporate uncertainty estimation, calibration, compartment-level localization, and systematic expert assessment, followed by prospective workflow studies to determine whether these systems can reduce workload, improve interobserver consistency, and provide reliable decision support in routine semen analysis.

## 5 Conclusion

This study systematically evaluated pretrained vision transformers, vision foundation models, and established CNN backbones for binary sperm morphology classification using a heterogeneous multi-source microscopy benchmark. Performance was strongly influenced by pretraining strategy, adaptation depth, and image source, with full fine-tuning generally improving pooled classification performance and selected light fine-tuning configurations remaining highly competitive. Although several transformer and foundation-model configurations achieved strong results, modern CNN baselines also performed well, indicating that no single architecture family was uniformly superior across all evaluation settings.

Source-specific and leave-one-source-out analyses further demonstrated that strong performance within a represented multi-source benchmark does not necessarily translate into reliable generalization to an unseen dataset. These findings support the continued development of AI-assisted sperm morphology systems as decision-support tools for improving consistency, quality assurance, and workflow efficiency within standardized semen analysis, rather than replacing expert assessment. Future work should prioritize prospective, multi-center validation, finer-grained and multi-label morphology modeling, calibrated uncertainty estimation, and integration with broader digital andrology workflows to establish clinical robustness and real-world utility.

## Data Availability

Publicly available datasets were analyzed in this study. This data can be found here: The Sperm Morphology Image Dataset (SMIDS) is available at https://data.mendeley.com/datasets/6xvdhc9fyb/1, the Human Sperm Head Morphology dataset (HuSHeM) is available at https://data.mendeley.com/datasets/tt3yj2pf38/3, and the HiLabSpermMorpho dataset is available at https://github.com/Yildiz-Hi-Lab/Hi-LabSpermMorpho. The code for experimental evaluation is available @ https://github.com/rawan-alsaad/Vision_Transformers_for_Sperm_Morphology.
