# Supplementary material for: Evaluation of vision transformers and vision foundation models for sperm morphology analysis
Source: Front Reprod Health. 2026 Jul 23;8:1883326. doi: 10.3389/frph.2026.1883326 (PMC13442368; doi:10.3389/frph.2026.1883326)
Supplement: Supplementary file 1 [file Supplementaryfile1.pdf]

Supplementary Material 1: Confusion Metrics of Transformer Models

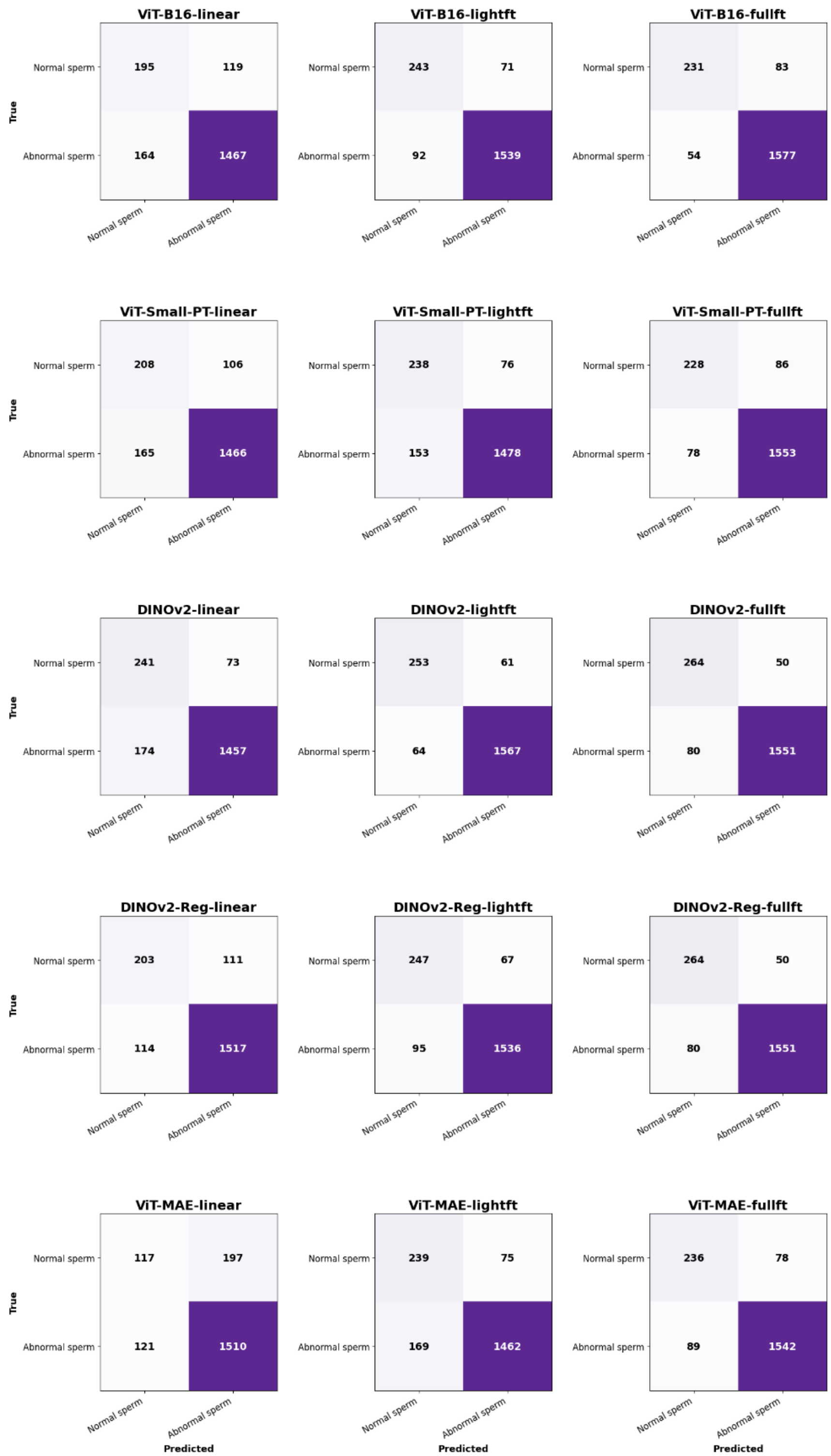

Figure S1A. Pooled confusion metrics of transformer models (Part 1)

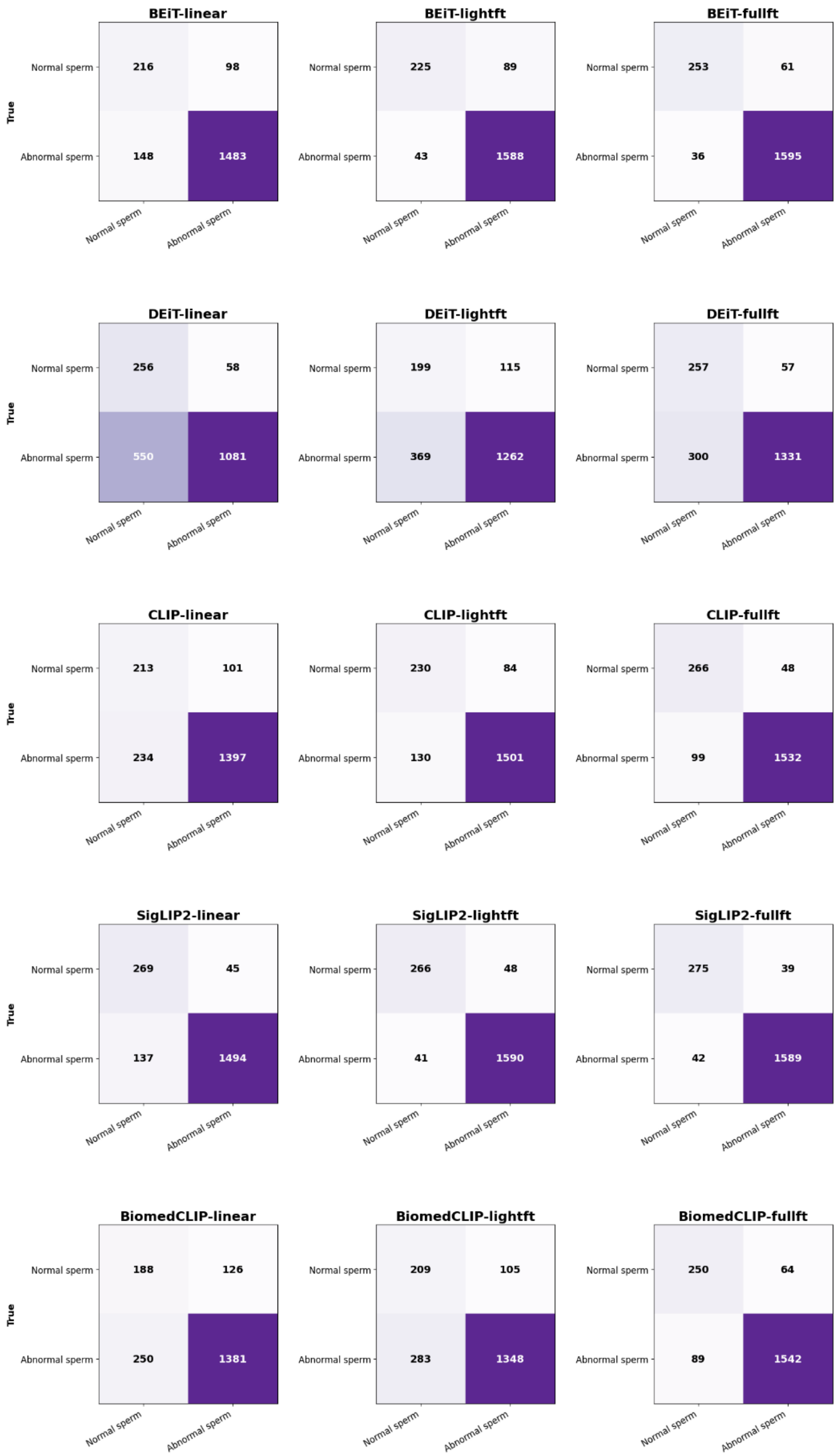

Figure S1B. Pooled confusion metrics of transformer models (Part 2)

Supplementary Material 2: Results of External Cross-Dataset Generalization Using Leave-One-Source-Out Validation

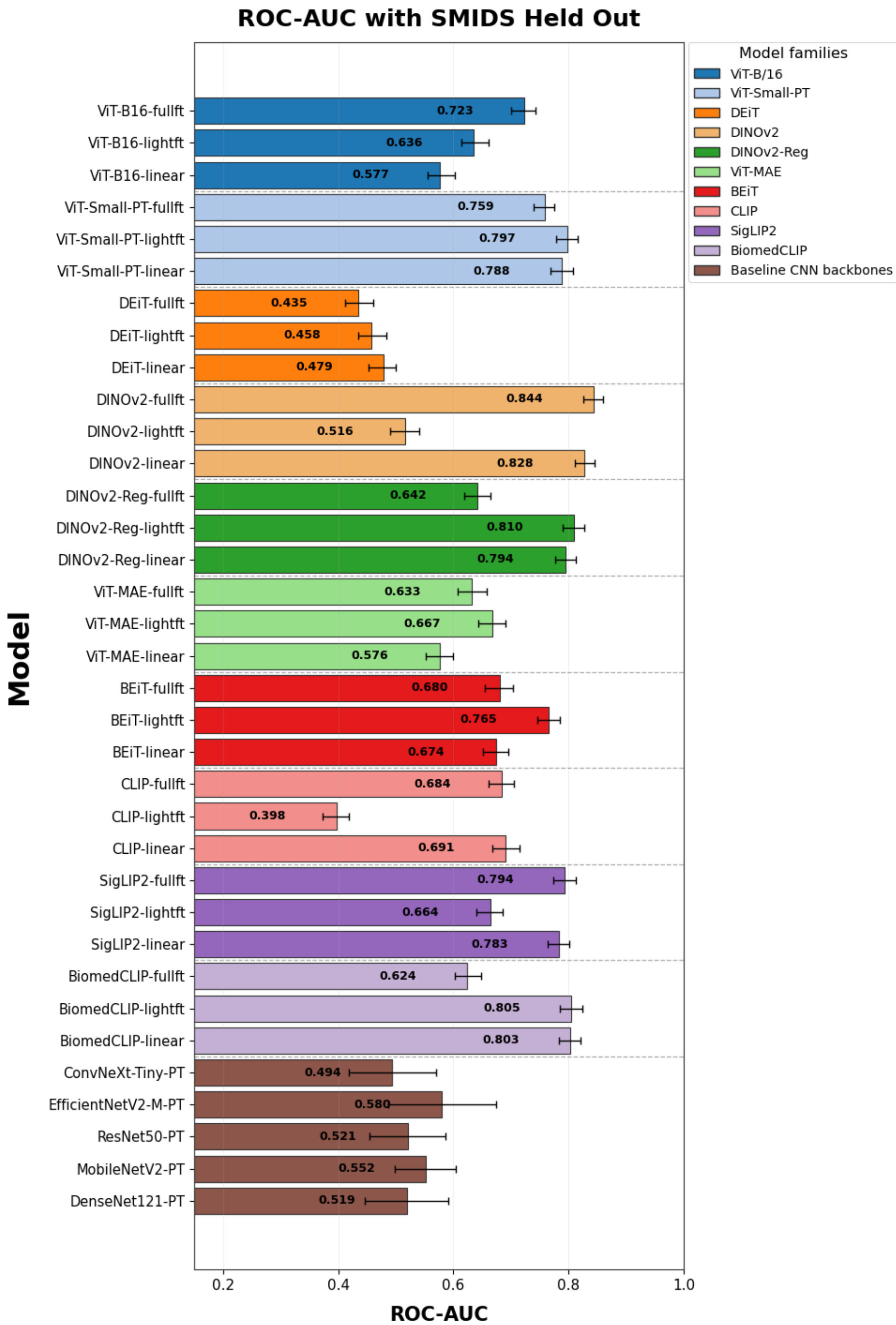

Figure S2A. ROC-AUC performance when models were trained on HuSHeM and HiLabSpermMorpho and evaluated on the completely held-out SMIDS dataset. Error bars show bootstrap 95% confidence intervals for transformer models and 95% confidence intervals across five seeds for CNN baselines.

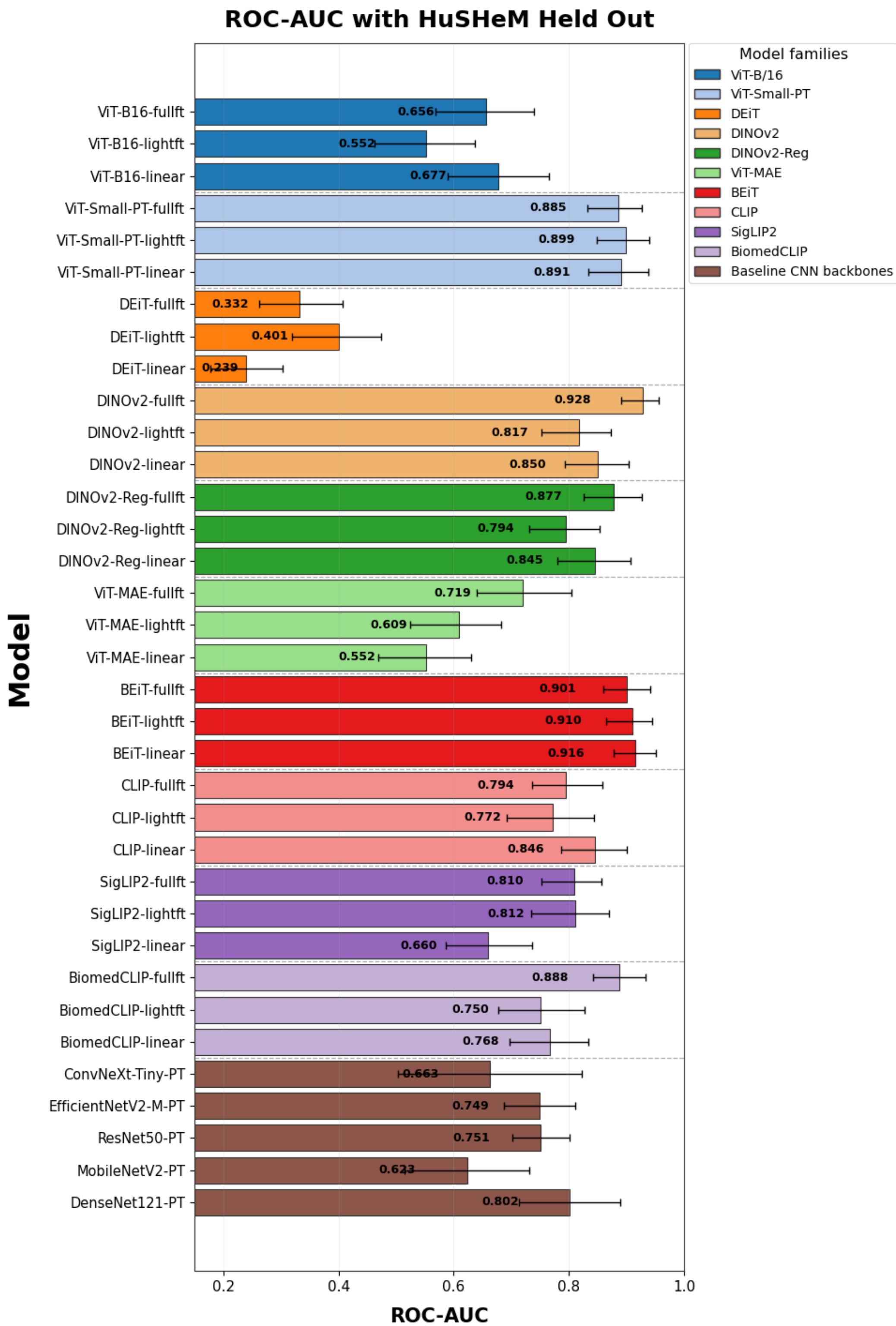

Figure S2B. ROC-AUC performance when models were trained on SMIDS and HiLabSpermMorpho and evaluated on the completely held-out HuSHeM dataset. Error bars show bootstrap 95% confidence intervals for transformer models and 95% confidence intervals across five

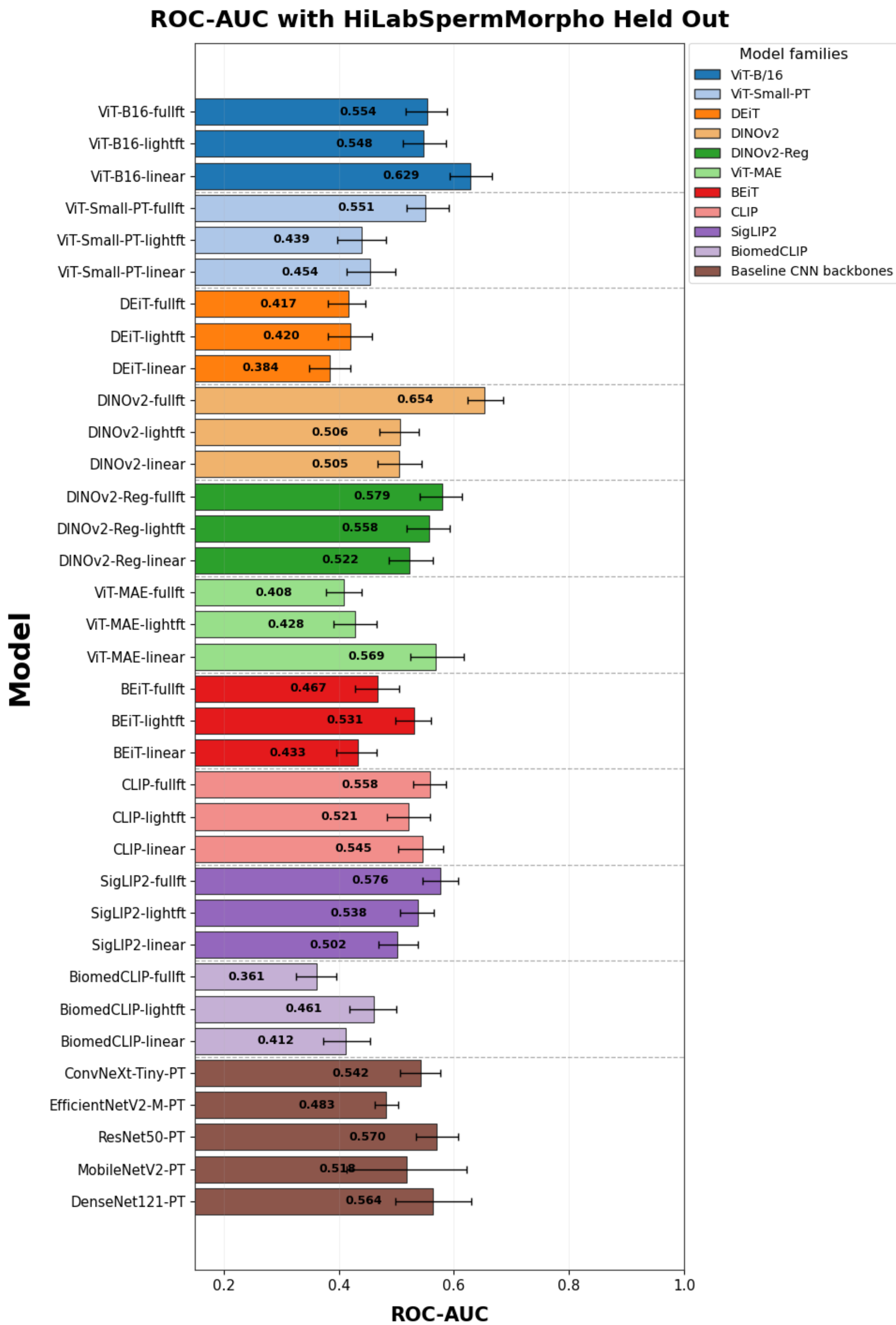

Figure S2C. ROC-AUC performance when models were trained on SMIDS and HuSHeM and evaluated on the completely held-out HiLabSpermMorpho dataset. Error bars show bootstrap 95% confidence intervals for transformer models and 95% confidence intervals across five seeds for CNN baselines.

Supplementary Material 3: Explainability Examples for Transformer Models

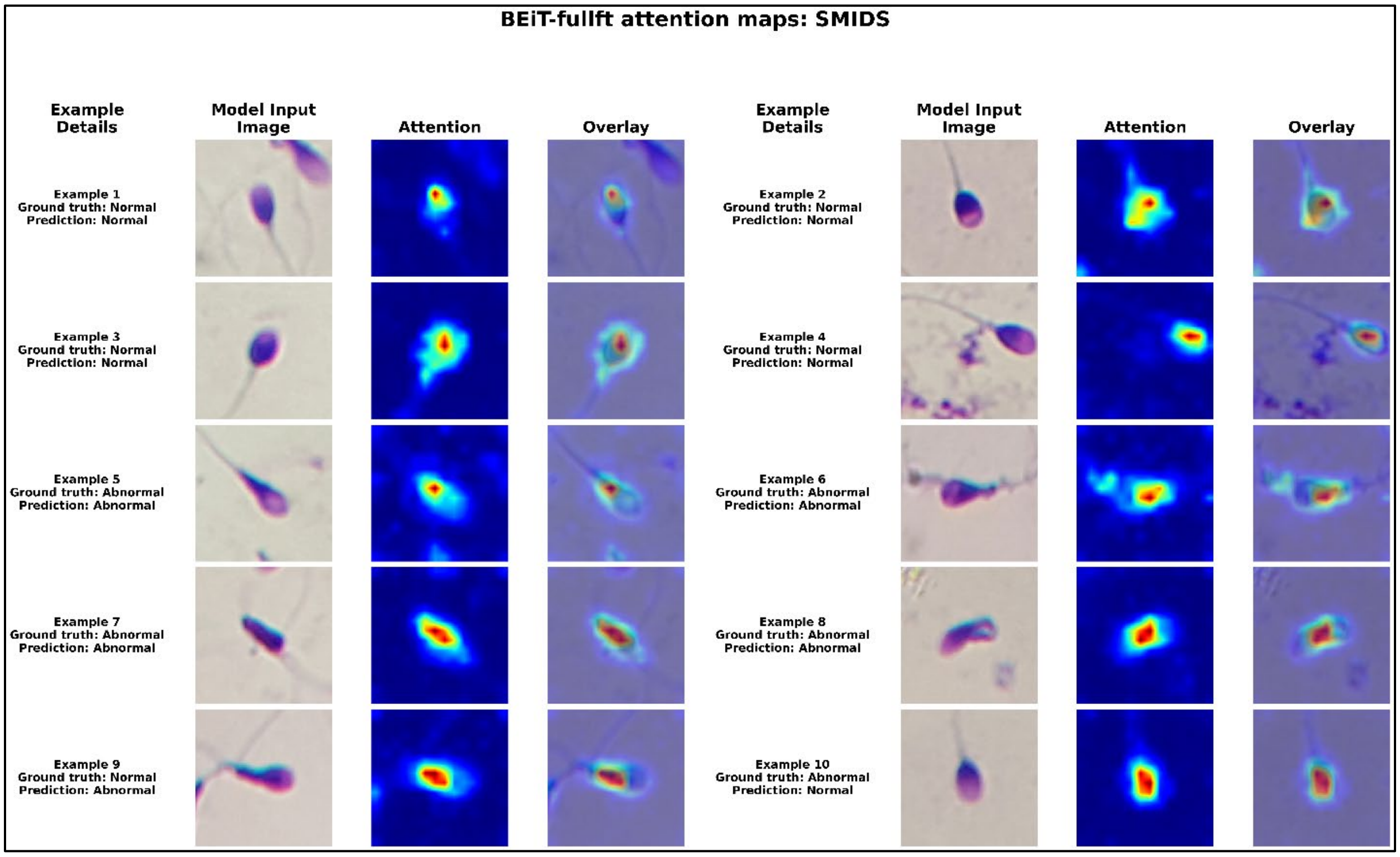

Supplementary Figure 3A. BEiT-fullft explainability examples for the SMIDS dataset. Ten representative test examples are shown, including normal and abnormal sperm images and misclassified cases. For each example, the ground-truth and predicted classes are provided alongside the model input image, the class-contrastive gradient-weighted attention map, and the corresponding overlay. Warmer colors indicate regions contributing more strongly to the model's prediction.

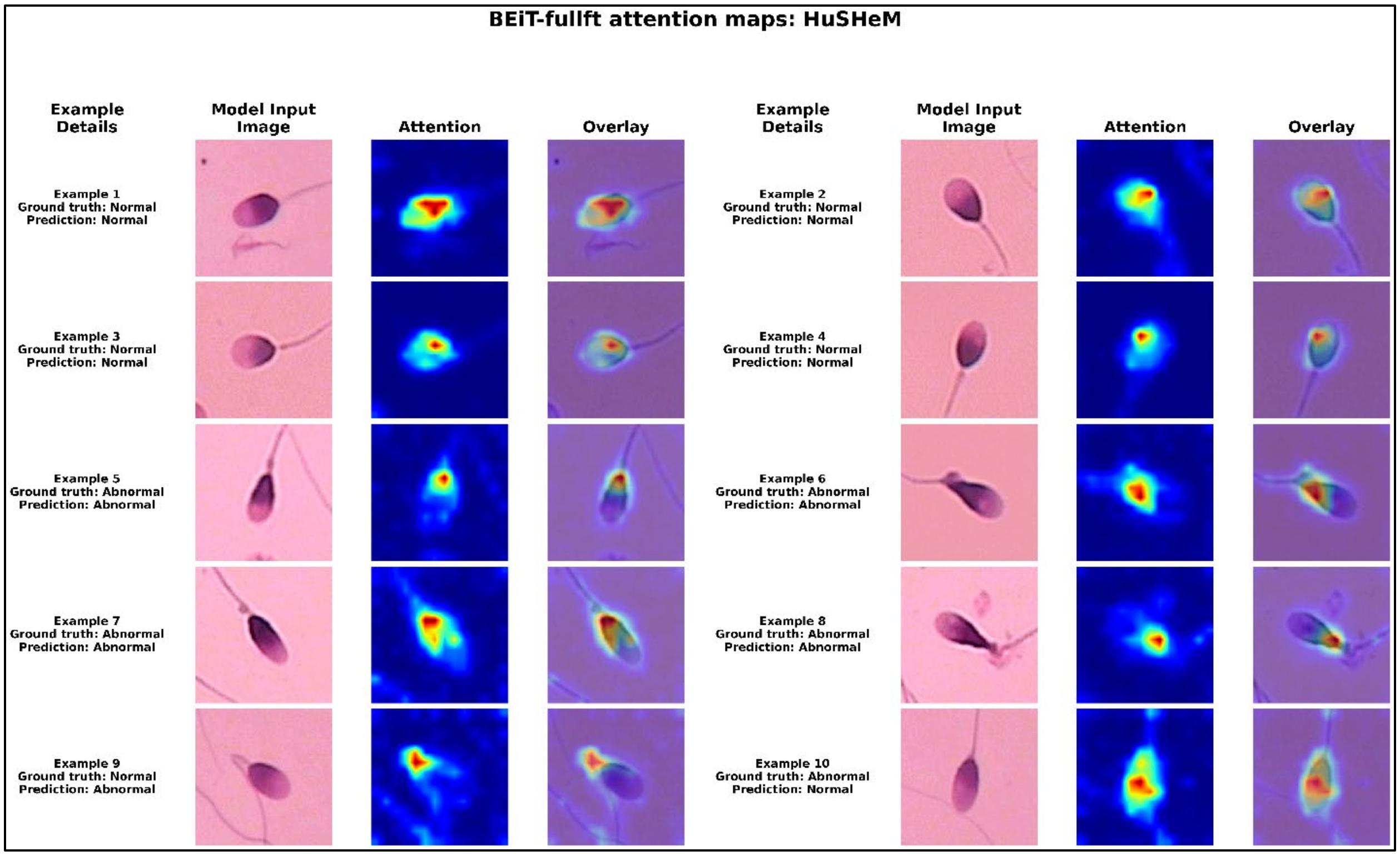

Supplementary Figure 3B. BEiT-fullft explainability examples for the HuSHeM dataset. Ten representative test examples are shown, including normal and abnormal sperm images and misclassified cases. For each example, the ground-truth and predicted classes are provided alongside the model input image, the class-contrastive gradient-weighted attention map, and the corresponding overlay. Warmer colors indicate regions contributing more strongly to the model's prediction.

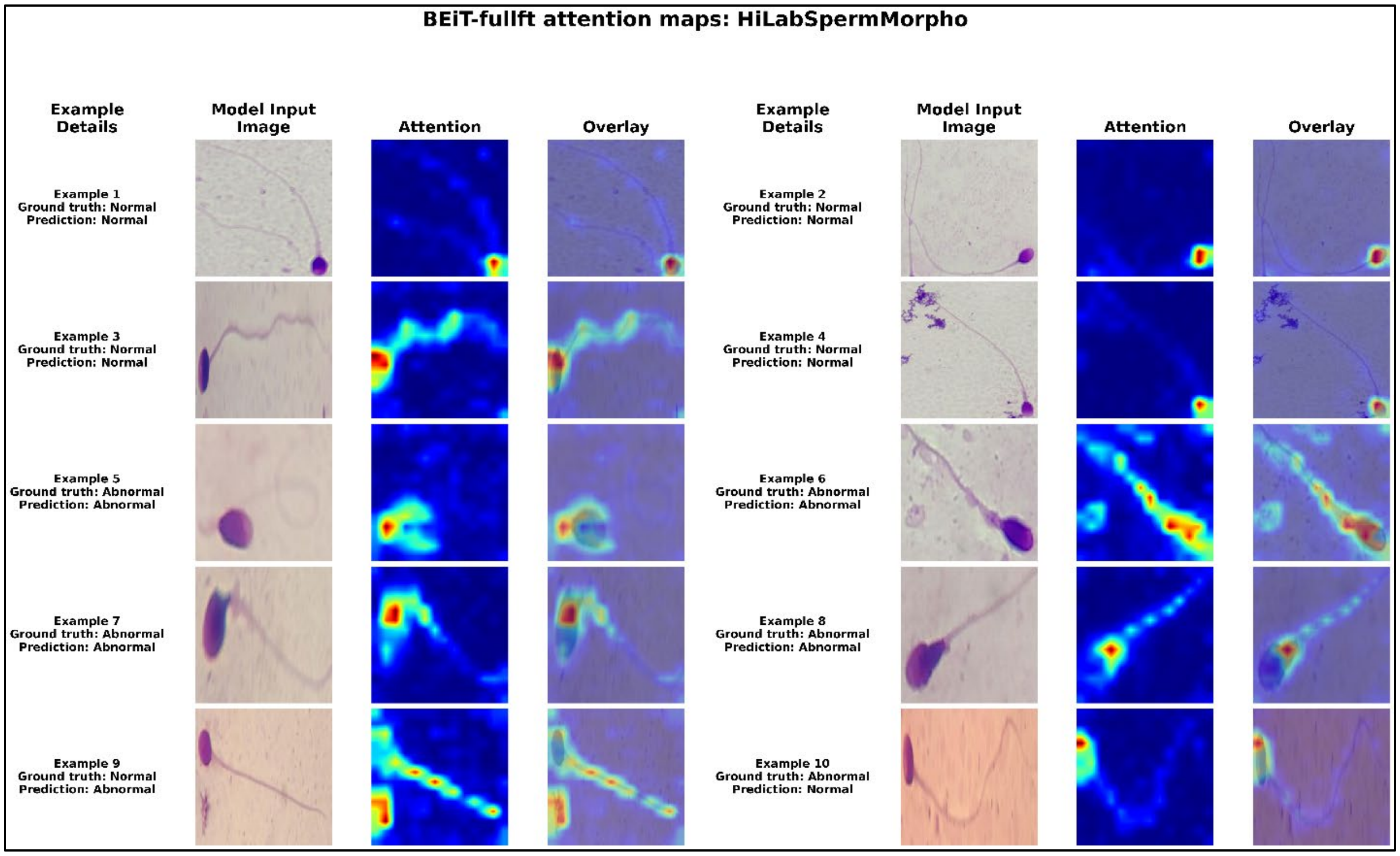

Supplementary Figure 3C. BEiT-fullft explainability examples for the HiLabSpermMorpho dataset. Ten representative test examples are shown, including normal and abnormal sperm images and misclassified cases. For each example, the ground-truth and predicted classes are provided alongside the model input image, the class-contrastive gradient-weighted attention map, and the corresponding overlay. Warmer colors indicate regions contributing more strongly to the model’s prediction.

Supplementary Material 4: Explainability Examples for CNN-based Models

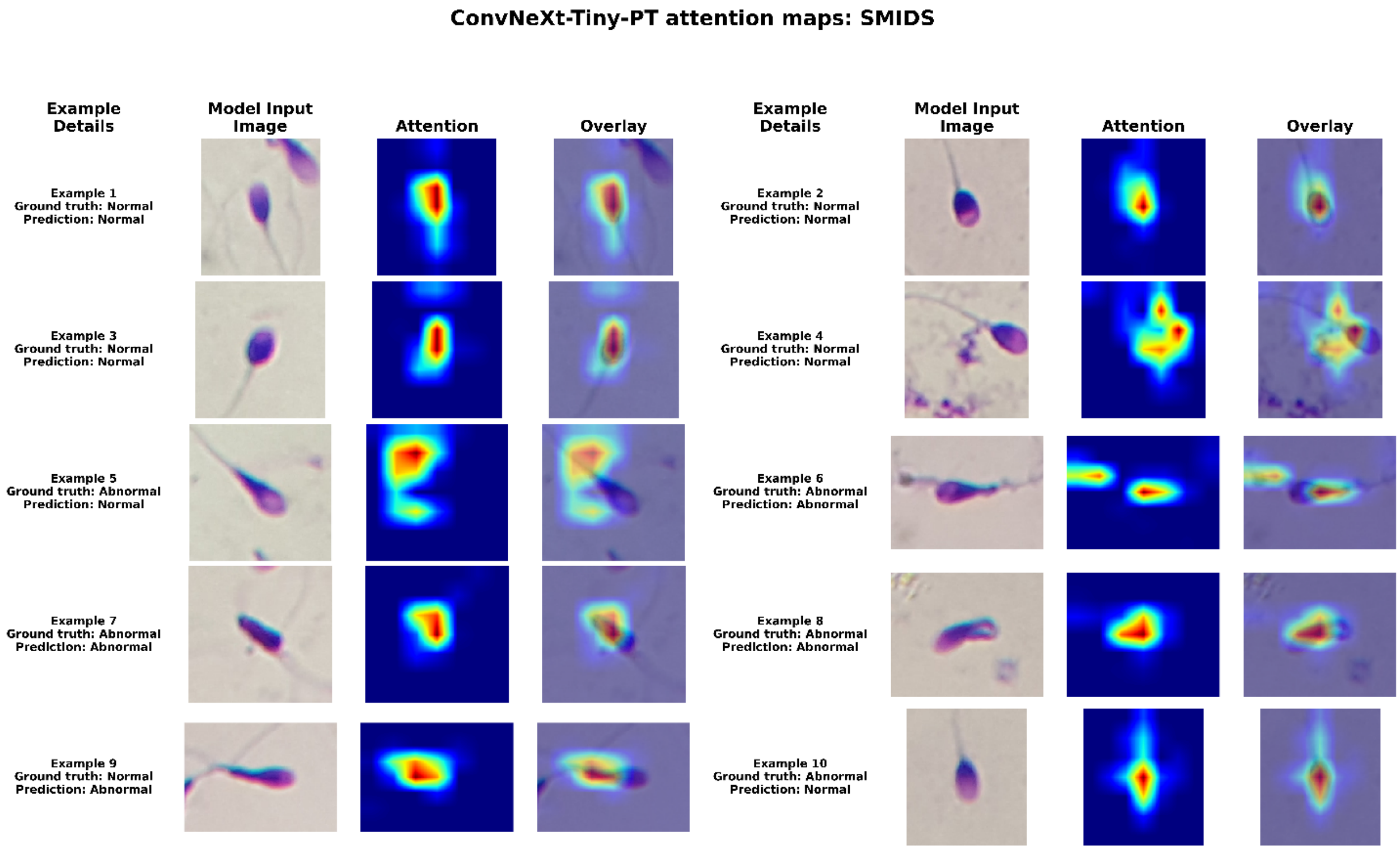

Supplementary Figure 4A. ConvNeXt-Tiny-PT explainability examples for the SMIDS dataset. Ten representative test examples are shown, including normal and abnormal sperm images and misclassified cases. For each example, the ground-truth and predicted classes are presented alongside the model input image, the class-contrastive Grad-CAM attribution map generated from the final ConvNeXt block, and the corresponding overlay. Warmer colors indicate image regions contributing more strongly to the model's prediction.

ConvNeXt-Tiny-PT attention maps: HuSHeM

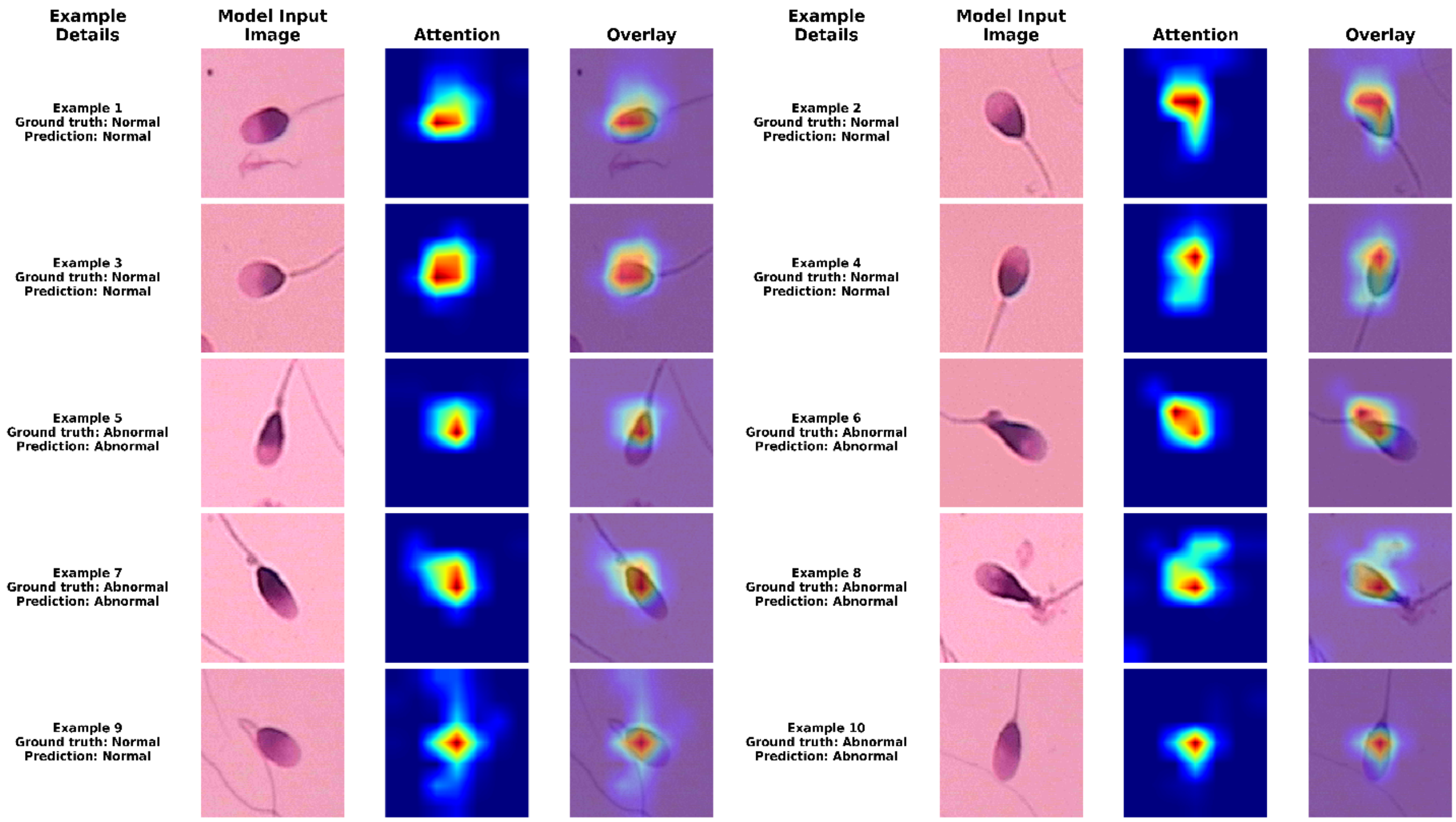

Supplementary Figure 4B. ConvNeXt-Tiny-PT explainability examples for the HuSHeM dataset. Ten representative test examples are shown, including normal and abnormal sperm images. For each example, the ground-truth and predicted classes are presented alongside the model input image, the class-contrastive Grad-CAM attribution map generated from the final ConvNeXt block, and the corresponding overlay. Warmer colors indicate image regions contributing more strongly to the model's prediction.

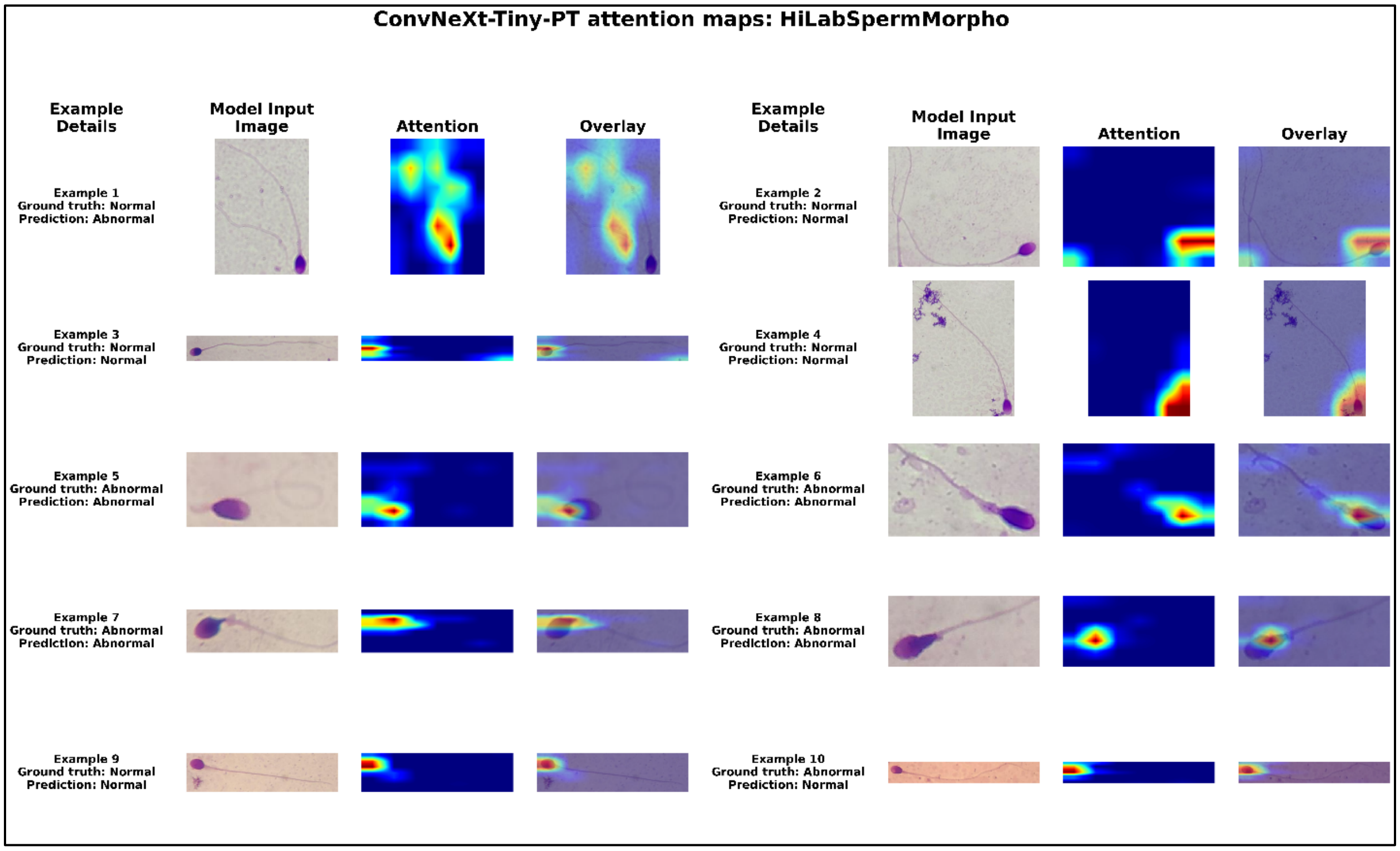

Supplementary Figure 3C. BEiT-fullft explainability examples for the HiLabSpermMorpho dataset. Ten representative test examples are shown, including normal and abnormal sperm images and misclassified cases where applicable. For each example, the ground-truth and predicted classes are provided alongside the model input image, the class-contrastive gradient-weighted attention map, and the corresponding overlay. Warmer colors indicate regions contributing more strongly to the model's prediction.
